# Quasi-Dry Electrochemical Mechanical Polishing Method of Additively Manufactured Parts

**DOI:** 10.3390/mi17101147

**Published:** 2026-09-30

**Authors:** Shihao Han, Zhiguang Sun, Xiaoyun Hu

**Affiliations:** College of Mechanical and Electrical Engineering, Nanjing University of Aeronautics and Astronautics, Nanjing 210016, China

**Keywords:** selective laser melting, TC4 titanium alloy, electrochemical mechanical polishing, surface roughness

## Abstract

Selective laser melting (SLM) of TC4 titanium alloy inevitably introduces surface defects including spheroidized particles, adhered powders, and microcracks, resulting in high roughness and poor flatness that prevent direct service. Electrochemical polishing (ECP) is suitable for complex curved surfaces, yet conventional immersion ECP consumes substantial acidic electrolytes, leading to waste and pollution. To overcome this, we propose a quasi-dry electrochemical mechanical polishing (Q-DECMP) method. Millimeter-scale abrasives serve as carriers, and an ethylene glycol-based NaCl electrolyte is coated onto the abrasive surfaces at an electrolyte-to-abrasive mass ratio of 1:20, existing only as a trace liquid film instead of immersing the workpiece. Owing to their high viscosity and capillary forces, a stable conductive liquid film forms on the abrasives. Since the abrasive size (2 mm) is larger than the microscopic peak-valley height of the SLM surface, the film-covered abrasives preferentially dissolve the peaks while the valleys remain unattacked, achieving selective leveling. Experiments performed at 200 V, 1 m/s linear velocity, and 6.5 mm interelectrode gap for 40 min show that Ra decreases from 6.89 to 0.77 μm (88.8% reduction) and material removal rate reaches 612.5 μg/min, outperforming pure mechanical polishing (Ra 4.98 μm, MRR 220 μg/min). With minimal electrolyte usage, this method reduces electrolyte consumption compared with conventional immersion electrochemical polishing, enabling efficient and low-loss finishing.

## 1. Introduction

Additive manufacturing technology achieves near-net shaping of parts through layer-by-layer deposition, breaking through the limitations of traditional subtractive manufacturing in terms of complex structural design, material utilization, and processing cycle, and is therefore widely applied in the preparation of small-batch complex components in fields such as aerospace, biomedical, energy equipment, and high-end manufacturing [1]. However, the metal additive manufacturing process typically involves rapid melting, solidification, and layer-by-layer bonding, which readily leads to surface morphology defects such as adhered powder, spheroidized particles, melt-track protrusions, and step effect on the formed surface [2]. These defects result in increased surface roughness, reduced geometric accuracy, diminished fatigue performance, and aggravated stress concentration, precluding the as-built parts from directly meeting the required in-service performance [3]. Therefore, the development of an efficient and precisely controllable post-processing polishing technology compatible with complex additively manufactured surfaces is essential for advancing the industrial adoption of additively manufactured metal parts [4].

Currently, a variety of surface post-treatment methods have been employed to improve the surface quality of additively manufactured metal components, including mechanical grinding, lapping, sandblasting, electrochemical polishing, and composite polishing. Mechanical grinding and lapping achieve high MRR through the abrasive cutting of surface asperities, making them suitable for geometrically regular external surfaces and areas with good tool accessibility. For example, Zhang et al. conducted mechanical polishing experiments and modeling studies on selective laser melting (SLM)-formed GH4169 alloy, reducing surface roughness from Ra 2.683 μm to Ra 0.8 μm [5]. However, such contact-based approaches are limited by the inherent need for direct tool–workpiece contact, which restricts their applicability to geometrically complex surfaces and hard-to-reach regions, while they also tend to induce periodic scratch patterns and mechanical damage. Sandblasting, a commonly used rough leveling method in industry, removes adhered powder and some surface protrusions through high-speed abrasive impact. Guerrero Vacas et al. compared the sandblasting effects of different abrasives on additively manufactured surfaces, showing that under optimal conditions, the surface roughness of H13 steel decreased from Ra 7.9 μm to Ra 3.6 μm, with roughness reductions for other alloys mainly ranging from 20% to 33% [6]. However, sandblasting is primarily suited for rough leveling and cannot readily produce high-precision polished surfaces. In contrast, chemical polishing and electrochemical polishing remove material through surface chemical reactions and anodic dissolution mechanisms, respectively. As non-contact processes, they impose significantly fewer constraints on workpiece geometry, demonstrating considerable application potential in the post-treatment of complex curved surfaces and difficult-to-machine materials. Cao et al. used a 15% by volume acetic acid aqueous solution as a weak acidic electrolyte, performing pure electrochemical polishing on DMLS 316L stainless steel with three printing layer thicknesses of 30 μm, 50 μm, and 60 μm at a constant temperature of 28 °C and a constant current density of 0.25 A/cm^2^. The original workpiece Sa ranged from 6.21 to 17.77 μm, with a maximum polishing time of 120 min, achieving a maximum roughness reduction of 73.8% under optimal conditions [7]. However, the polishing cycle of this electrolyte solution lasts up to 2 h, resulting in extremely low processing efficiency, and the polished surface still retains numerous deep pits and valleys. Additionally, acetic acid is corrosive, generating large amounts of acidic waste liquid during processing, leading to high environmental treatment costs.

To overcome the limitations of single mechanical or electrochemical machining, combined polishing technologies that integrate mechanical action with electrochemical action have attracted widespread attention. These technologies utilize mechanical action to break or renew the passive film on the surface, while simultaneously reducing material hardness through electrochemical dissolution, thereby improving machining efficiency and surface quality to a certain extent [8]. For example, Liao et al. applied chemical mechanical polishing to pre-ground TC4 titanium alloy for 25 min of polishing treatment, reducing the surface roughness from Ra 43.94 nm to Ra 0.661 nm, achieving a nanoscale ultra-smooth surface [9]. This result indicates that the synergy of mechanical and chemical actions can achieve extremely high surface quality, but it relies on pre-grinding treatment and is mainly suitable for flat surfaces with low initial roughness, making it difficult to directly address defects such as adhered powder, spheroidization, and large height differences on the original surface of additively manufactured parts. Krzak et al. proposed a combined PEMEC electrochemical and drag-grinding synchronous polishing process, using a phosphoric acid electrolyte with alumina abrasive media on 316 stainless steel with an initial surface (Ra = 12.2 μm). By systematically comparing three parameters—temperature, voltage, and drag rotation speed—the roughness was reduced to 0.18 μm within 30 min under optimal conditions of 60 °C, 12 V, and 100 rpm, shortening the processing time by more than 80% compared to pure drag grinding [10]. However, this process uses phosphoric acid as the electrolyte and fully immerses the abrasive grains in the electrolyte, resulting in high electrolyte consumption. Furthermore, the experimental samples in this study were milled to simulate rough surfaces, which had limited compatibility with the original as-printed SLM surfaces containing numerous micropores and semi-molten powder particles, and could not fully resolve the complete leveling of deep pores and spheroidized particles in the printed part. It is evident that although electrochemical mechanical combined machining can improve post-processing efficiency, traditional liquid-based systems still face bottlenecks such as high electrolyte usage, stray corrosion, difficulties in waste liquid treatment, and insufficient selectivity for removing protrusions. Therefore, how to reduce liquid electrolyte consumption while maintaining electrochemical dissolution efficiency and achieving localized, selective material removal is an urgent issue to be addressed for green post-processing in additive manufacturing.

In recent years, dry electrochemical polishing methods based on solid–liquid composite conductive media have provided new ideas for solving the above problems. These methods typically adsorb or fix a small amount of electrolyte within a solid medium, using the solid medium to transfer ions and establish local current pathways, thereby reducing the use of free liquid electrolyte. Liu et al. proposed a solid media electrochemical polishing method, using cation exchange resin as a solid electrolyte to treat SLM 316L stainless steel parts. By regulating the MRR in stages, the surface roughness was reduced from 10 μm to 0.75 μm, an improvement rate of over 92% [11]. Liu et al. used macroporous ion exchange resin adsorbed with 40% sulfuric–phosphoric mixed electrolyte as the solid medium, with a pulsed power supply regulating the process, to treat the original as-built side surface of SLM-formed 316L stainless steel (initial average Sa = 17.16 μm). Through response surface optimization, the optimal process was obtained: 3.65 wt% sulfuric acid, 50 V voltage, 90% duty cycle, and 150 rpm rotation speed, reducing Sa to 3.09 μm in just 30 min, a roughness reduction rate of 82% [12]. However, ion exchange resins are costly and prone to blockage by wear debris, leading to short service life; additionally, the electrolyte system is still primarily based on weak acids, posing corrosion risks. Bai et al. used polymer spherical solid media adsorbed with a trace amount of dilute sulfuric acid to conduct dry electrochemical mechanical polishing experiments on SLM-formed 316L stainless steel; under the optimal conditions (combined polishing at 50 V for 6 h followed by 1 h of pure mechanical fine polishing), the surface roughness (Ra) of the workpiece decreased from 8.72 μm to 0.75 μm, demonstrating the leveling capability of the quasi-dry combined polishing process [13]. This process required a single polishing cycle as long as 6 h, with material removal efficiency needing improvement. Furthermore, the polishing medium still relied on an acid-based electrolyte, and a clean mirror surface could not be obtained through electromechanical combined treatment alone; an additional 1 h of pure mechanical fine polishing was necessary, resulting in a complex overall processing procedure [14].

Electrochemical polishing methods based on solid–liquid composite conductive media fundamentally reduce the generation of liquid waste, demonstrating the potential of solid media electrochemical polishing for surface finishing in additive manufacturing. However, ion exchange resins are relatively expensive and are prone to blockage by wear debris, oxides, and reaction products during processing, leading to a decline in ion transport capacity and process stability. Additionally, the processes for regeneration, maintenance, and parameter adjustment of the resin media are relatively complex, hindering low-cost, large-scale industrial application.

Based on the above issues, this paper proposes a quasi-dry electrochemical mechanical polishing (Q-DECMP) for efficient and low-cost surface finishing of SLM-TC4 titanium alloy. This process uses 2 mm alumina abrasive grains as the electrolyte carrier, leveraging the high viscosity and low volatility of ethylene glycol to stably attach a small amount of NaCl-ethylene glycol (EG; Sinopharm Chemical Reagent Co., Ltd., Shanghai, China) electrolyte to the surface of the abrasive grains, forming a conductive liquid film and liquid bridges in the contact area between the abrasive grains and the workpiece. Since the size of the abrasive grains is significantly larger than the characteristic dimensions of the peaks and valleys on the surface produced by SLM, the abrasive grains can only bridge over and contact the peak regions. The electrolyte film carried on the surface of the abrasive grains forms a local electrochemical circuit at the contacted peaks, inducing anodic dissolution and thereby achieving the preferential removal of the peak material. In the valley regions, neither the abrasive grains nor the liquid film can make contact, so these areas are subjected to neither electrochemical action nor any mechanical force. This localization mechanism based on scale differences strictly confines material removal to the peak regions, ultimately achieving localized surface leveling. This paper further studies the electrochemical behavior of the NaCl-EG system and analyzes the effects of processing parameters such as applied voltage, mass ratio of electrolyte to abrasive grains, and linear velocity on surface roughness, MRR, and morphological evolution, providing an efficient and low-loss route for electrochemical post-processing of additively manufactured metal parts, with reduced electrolyte consumption compared with conventional immersion electrochemical polishing.

## 2. Principles of Q-DECMP

**Figure 1:**
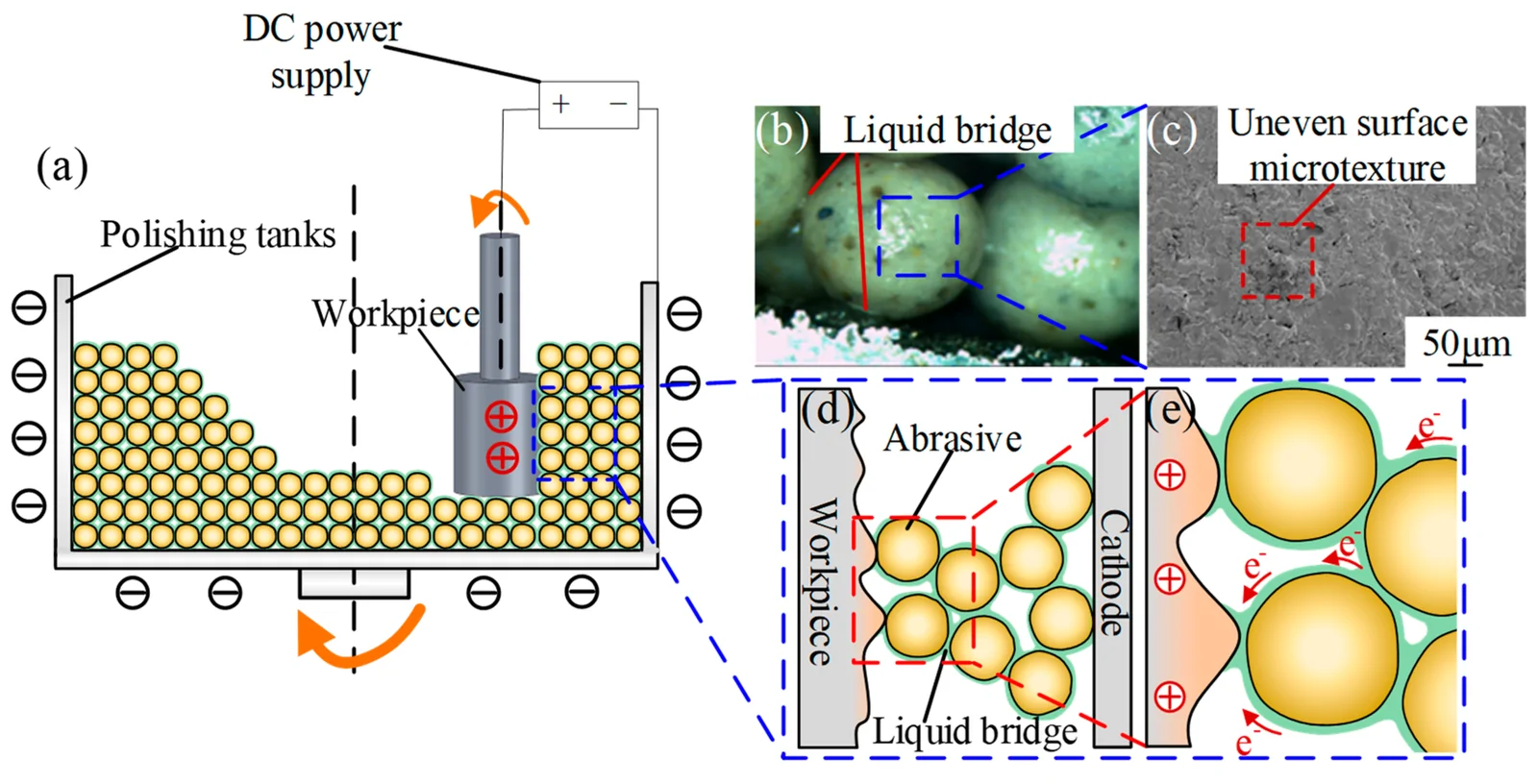
Q-DECMP process principle and local material removal mechanism: (**a**) Schematic diagram of Q-DECMP device and processing principle; (**b**) electrolyte film on the surface of alumina abrasive grains and liquid bridge between abrasive grains; (**c**) SEM morphology of abrasive grain surface; (**d**) local contact state between abrasive grains, electrolyte and workpiece; (**e**) magnified view of (**d**): liquid film and conductive channel of the liquid bridge.

The principle of the Q-DECMP process is shown in Figure 1a. During processing, the SLM-TC4 titanium alloy workpiece is connected to the anode of a DC power supply, while a cylindrical groove-shaped conductive container is connected to the cathode and serves as a polishing tank to hold alumina abrasive grains and a small amount of NaCl-EG electrolyte. Alumina abrasive grains with an average diameter of approximately 2 mm are used as the electrolyte carrier and mechanical action medium. When a small amount of NaCl-EG electrolyte is added to the particle system, due to the high viscosity and low volatility of ethylene glycol, the electrolyte can stably cover the surface of the particles under the action of surface tension, forming a conductive liquid film. Meanwhile, at the local contact areas between particles and between particles and the workpiece, the electrolyte forms liquid bridges under capillary force, as shown in Figure 1b. This liquid film and liquid bridges provide ion transport channels for electrochemical reactions, enabling the establishment of a current loop in the local area. Alumina abrasive grains are non-conductive, and the numerous concave and convex microstructures distributed on their surface facilitate electrolyte adhesion (as shown in Figure 1c). Compared with a flat morphology, this rough and uneven morphology can increase the contact area between the electrolyte and the abrasive particle surface, enabling the electrolyte to adhere stably to the surface and be retained in the depressions, thereby continuously forming an electrolyte liquid bridge between the abrasive particle and the workpiece micro-asperities and providing a stable medium for the electrochemical reaction.

Due to the very low electrolyte ratio, the liquid medium does not fill the entire machining gap but is instead adsorbed as an extremely thin liquid film on the surface of the abrasive grains and around the workpiece peak areas. Driven by surface tension, localized “liquid bridges” are formed in the micro–nano gaps around the contact points between particles and peaks. Meanwhile, adjacent particles are also connected through the extremely thin liquid film, as are the particles and the electrode surface, forming narrow and continuously conductive ion migration pathways (as shown in Figure 1d,e). Based on this contact state, a proposed mechanism for selective peak removal is described as follows. The peak regions are preferentially in contact with the conductive liquid film and liquid bridges, so anodic dissolution mainly occurs at these regions, whereas the valley regions remain largely unattacked because no reliable conductive path is established there. As the process continues, the surface peaks are removed layer by layer, and the height difference between peaks and valleys gradually decreases, ultimately achieving selective leveling of the rough surface.

## 3. Experimental Materials and Methods

### 3.1. Materials and Characterization

**Figure 2:**
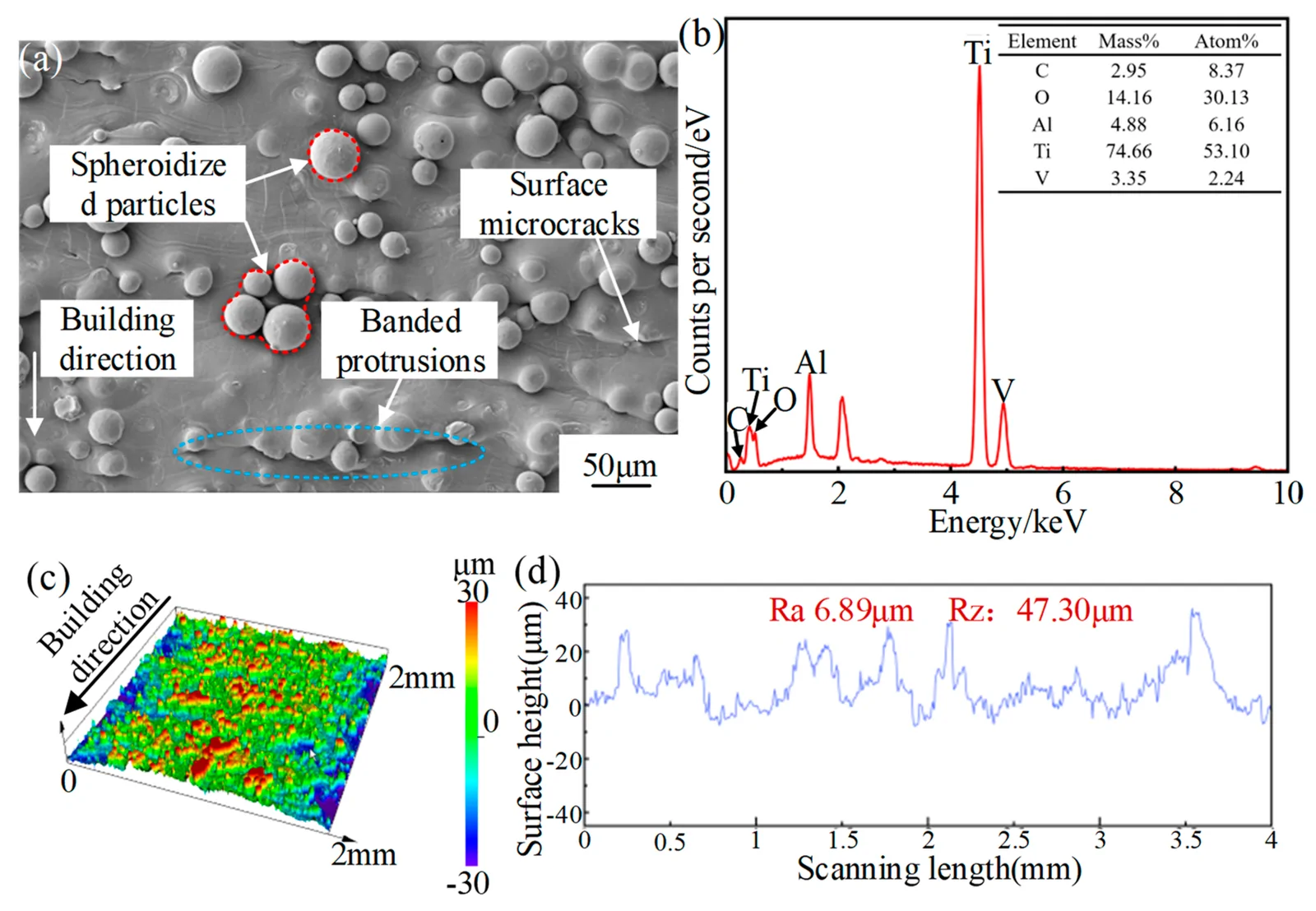
Initial surface characteristics of SLM-TC4 titanium alloy: (**a**) SEM morphology; (**b**) EDS analysis results; (**c**) three-dimensional topography; (**d**) roughness profile. The values in (**d**) are the mean values, and the corresponding standard deviations are given in Section 3.1 (*n* = 3).

The experimental specimen was a cylindrical TC4 titanium alloy sample fabricated by SLM, with dimensions of Φ15 mm × 7 mm. The TC4 powder characteristics and the main SLM process parameters are listed in Table 1. To characterize its initial surface condition, scanning electron microscopy (SEM, ZEISS Sigma 360, Oberkochen, Germany), energy-dispersive spectroscopy (EDS, attached to the SEM), and laser confocal microscopy (LEXT OLS5100, Olympus Corporation, Tokyo, Japan) were used to examine the surface morphology, elemental composition, and three-dimensional profile. As shown in Figure 2a, the initial surface of the SLM-TC4 titanium alloy was relatively rough, exhibiting numerous spheroidized particles of varying sizes, banded protrusions distributed along the build direction, and microcracks. Some spheroidized particles adhered to one another and were attached to the banded protrusion areas, further increasing the local height differences on the surface. The spheroidized particles were mainly associated with the adhesion of incompletely melted powder, molten metal spatter, and the balling effect, while the banded protrusions likely originated from the melt track protrusions and step effect during the layer-by-layer building process. These forming defects collectively result in a markedly uneven surface profile with pronounced undulations [15].

To analyze the elemental composition of the initial surface, EDS mapping was performed on the specimen surface, as shown in Figure 2b. The initial surface mainly consists of Ti, Al, and V, consistent with the matrix composition of TC4 titanium alloy. Meanwhile, a certain amount of O and a small amount of C were detected on the surface. The O content is 14.16 wt%, which may be primarily attributed to the oxide layer on the specimen surface and oxidation products formed during the SLM process. The C may originate from environmental contamination or residues from specimen preparation. The three-dimensional morphology shown in Figure 2c further indicates that the initial surface exhibits pronounced peak-to-valley undulations and banded protrusions, with local peaks and valleys alternating in distribution, corresponding to the spheroidized particles and banded protrusions observed in the SEM images. Roughness measurements were performed at five different positions along the direction perpendicular to the build direction, yielding an initial surface average roughness Ra of 6.89 ± 1.2 μm (*n* = 3). The roughness profile is shown in Figure 2d, with an Rz of 47.3 ± 6.9 μm (*n* = 3) indicating a substantial peak-to-valley height difference on the initial surface.

In summary, the initial surface of the SLM-TC4 titanium alloy exhibits pronounced spheroidized particles, banded protrusions, microcracks, and severe peak-to-valley undulations, with poor surface roughness and flatness, indicating that further post-processing is required to meet surface quality requirements.

### 3.2. Electrolyte Preparation

**Figure 3:**
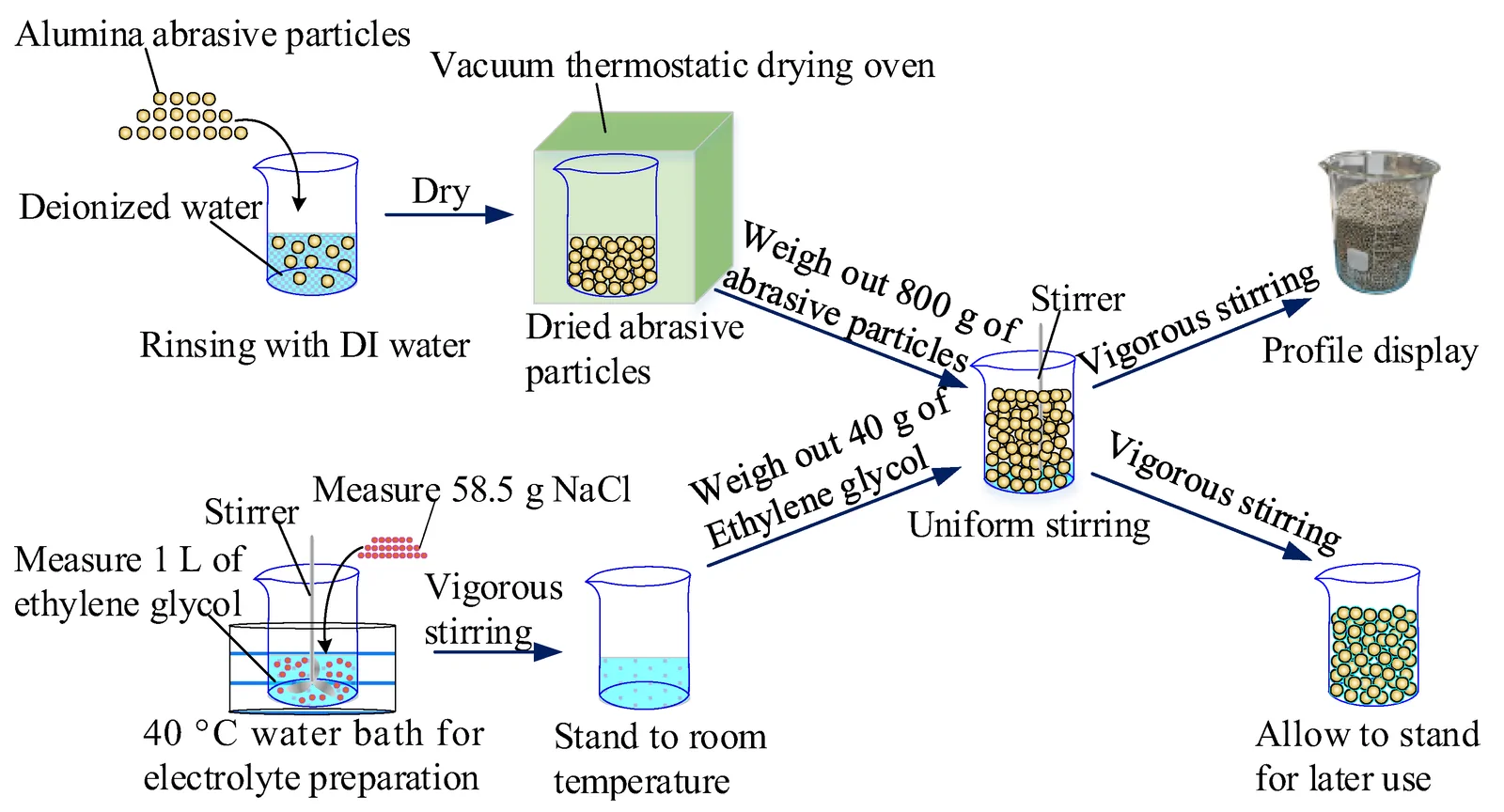
Electrolyte preparation steps.

The electrolyte is a NaCl-EG system, with the specific formulation shown in Table 2. Due to the high viscosity of ethylene glycol and the slow diffusion of NaCl, stirring should not be too rapid when preparing the NaCl-EG electrolyte. The specific preparation steps are shown in Figure 3: slowly add 58.5 g of analytically pure NaCl into 1.0 L of EG, stir with a stirrer at 40 °C at a speed of 300–400 r/min until the NaCl is completely dissolved and the solution is clear and transparent. Then allow it to stand for 10 min to fully homogenize the solution, and set it aside for later use [16]. The electrolyte was prepared by dissolving analytical-grade NaCl in ethylene glycol, with a small amount of additive to improve the stability of the liquid film. Wash the 2 mm alumina abrasive grains with deionized water (Suzhou Chunlu Water Treatment Technology Co., Ltd., Kunshan, China) and then place them in a vacuum thermostatic drying oven to completely remove moisture. Weigh 800 g of alumina abrasive grains and 40 g of NaCl-EG solution, and mix thoroughly with a stirrer at low speed.

### 3.3. Electrochemical Testing

**Figure 4:**
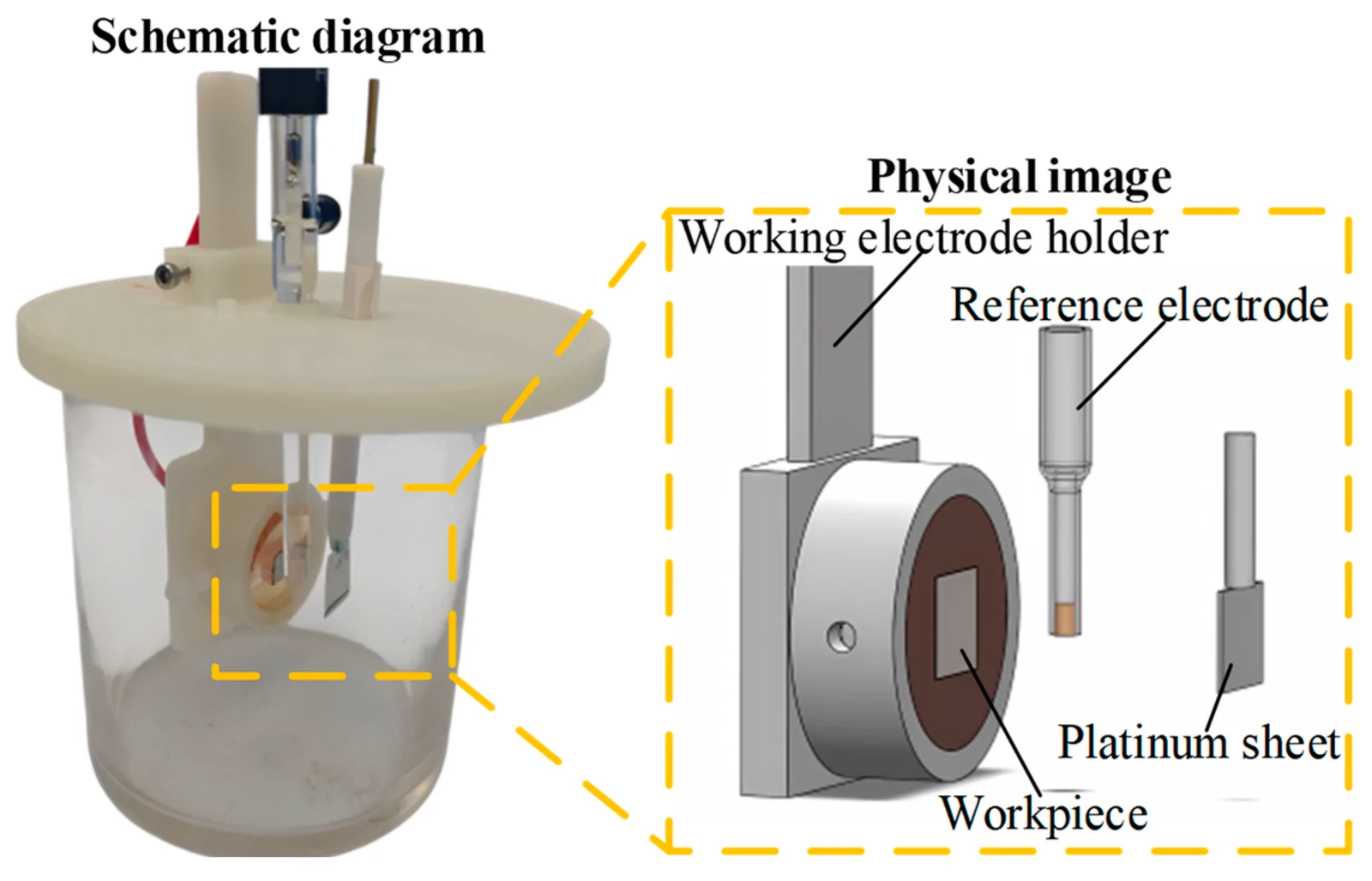
Schematic diagram of the three-electrode electrochemical testing setup and electrode assembly for TC4 titanium alloy.

To evaluate the electrochemical reaction characteristics of TC4 titanium alloy in a NaCl-EG electrolyte system and to select a suitable electrolyte system for subsequent Q-DECMP experiments, three solutions—0.5 mol/L NaCl-EG, 1 mol/L NaCl-EG, and 1 mol/L NaCl-H_2_O—were selected for comparative testing in this study. Among these, the 0.5 mol/L NaCl-EG and 1 mol/L NaCl-EG solutions were used to analyze the effect of NaCl concentration on the conductivity, passivation behavior, and anodic reactivity of the ethylene glycol-based electrolyte; the 1 mol/L NaCl-H_2_O solution served as an aqueous electrolyte control for comparing the differences in electrochemical response and corrosion morphology on the TC4 surface under different solvent systems. Electrochemical tests were conducted using a three-electrode setup (the electrochemical test setup and schematic diagram are shown in Figure 4). The TC4 specimen served as the working electrode, a platinum sheet as the counter electrode, and a saturated calomel electrode (SCE, LICHEN, Changsha, China) as the reference electrode. Before testing, all areas of the working electrode except the test surface were insulated and encapsulated, with an effective exposed area of 1 cm^2^ (10 mm × 10 mm). The test temperature was controlled at 25 ± 1 °C, and each electrolyte condition was tested three times to ensure result reliability.

**Figure 5:**
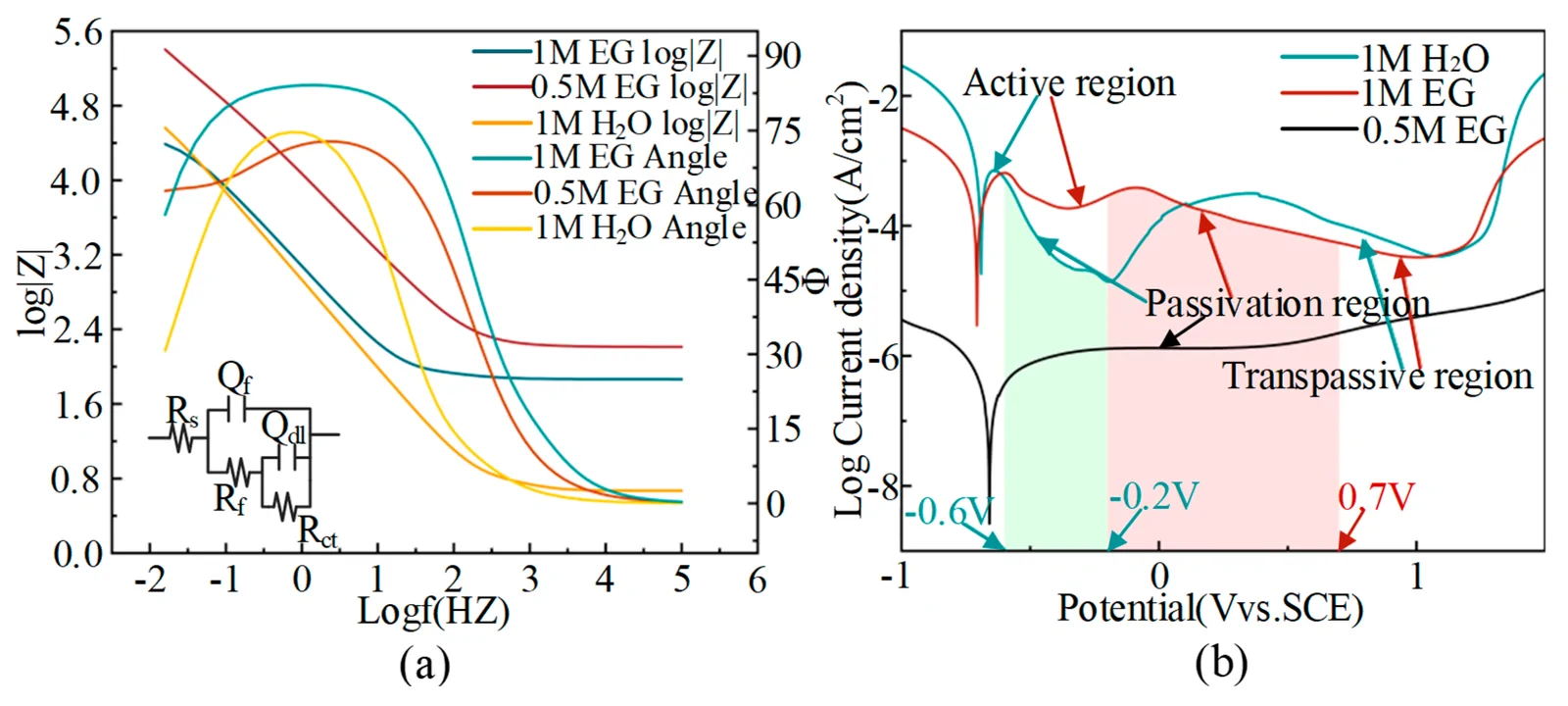
Fitted EIS total spectra and polarization curves from electrochemical tests: (**a**) fitted EIS total spectra; (**b**) polarization curves of the three solutions. In (**b**), the light green shaded region (−0.6 to −0.2 V) indicates the passivation region of the H_2_O system (1 M H_2_O), and the light red shaded region (−0.2 to 0.7 V) indicates the passivation region of the EG system (1 M EG). M = mol/L; EG = NaCl–EG; H_2_O = NaCl–H_2_O.

The test procedure sequentially included open circuit potential (OCP) monitoring, electrochemical impedance spectroscopy (EIS) testing, and potentiodynamic polarization testing. The OCP was first recorded for approximately 2 h until the potential change stabilized, ensuring a relatively stable electrode/electrolyte interface. Subsequently, EIS testing was performed at the stable OCP, applying a sinusoidal perturbation signal with an amplitude of 10 mV over a frequency range of 10^5^–10^−2^ Hz. The EIS spectra were fitted employing an equivalent circuit of $R_{s}(Q_{f}(R_{f}(Q_{dl}R_{ct})))$, as shown in Figure 5a, where $R_{s}$ denotes the solution resistance, $Q_{f}R_{f}$ accounts for the surface oxide film, and $Q_{dl}R_{ct}$ corresponds to the double-layer capacitance and charge-transfer resistance at the substrate interface [17]. CPEs were adopted in place of ideal capacitors to address the frequency dispersion induced by surface roughness. Fitting was performed using Zsimpwin software 3.6 based on complex nonlinear least-squares (CNLS) regression. The chi-squared value ($\chi^{2}$) remained below 8 × 10^−4^ for all spectra, confirming that the selected circuit provides a valid representation of the interfacial electrochemical response. Finally, Potentiodynamic polarization measurements were performed at a scan rate of 2 mV/s over a potential range from −1.0 to 1.5 V vs. SCE to investigate the anodic dissolution, passivation, and localized corrosion susceptibility of TC4 titanium alloy in different electrolytes.

### 3.4. Experimental Details

The Q-DECMP experimental setup mainly consists of a DC power supply (eTM-40010PC, eTOMMENS, Dongguan, Guangdong Province, China), a workpiece drive motor, a cathode polishing tank drive motor, and a 316L stainless steel cylindrical tank used as the polishing container, where the 316L stainless steel polishing tank serves as the tool cathode. Before the experiment, the SLM-TC4 titanium alloy sample was ultrasonically cleaned in anhydrous ethanol (Sinopharm Chemical Reagent Co., Ltd., Shanghai, China) for 10 min to remove surface oil stains and loosely attached particles, and then dried with compressed air. The cleaned sample was fixed onto the anode fixture, and the polishing tank was filled with alumina abrasive grains and a NaCl-EG electrolyte solution at a specified ratio.

During the machining process, the workpiece and the polishing tank were independently driven by separate motors and rotated in opposite directions, causing the abrasive grains to continuously roll, press, and rub against the sidewall surface of the workpiece, as shown in Figure 1a. The DC power supply applied a set voltage between the workpiece and the polishing tank, establishing conductive pathways through the liquid film on the abrasive particle surfaces and local liquid bridges at the workpiece asperity peaks, thereby realizing a synergistic effect of electrochemical dissolution and mechanical-assisted removal. The detailed experimental parameters are given in Table 3.

### 3.5. Detection Method

A handheld roughness tester (TR200, Guangzhou Junda Instrument and Meter Co., Ltd., Guangzhou, China) was used to measure the surface roughness Ra of the cylindrical sidewall of the specimen. Considering the initial surface roughness level of the specimen, the sampling length was set to 0.8 mm, and the evaluation length consisted of 5 consecutive sampling lengths, i.e., 4 mm. To enhance the representativeness of the test results, five different positions on the cylindrical sidewall of each specimen were selected for measurement, and the average value was calculated as the surface roughness result for that specimen. Three repeated experiments were conducted for each set of process parameters, and the final result was taken as the average. The mass of the specimen before and after processing was measured using a precision electronic balance with an accuracy of 0.001 g. The material removal amount was calculated based on the mass difference before and after processing, and combined with the processing time, the material removal rate (MRR) was obtained.

The surface morphology of the specimens before and after processing was observed using a scanning electron microscope (SEM, ZEISS Sigma 360, Oberkochen, Germany) to analyze the removal of spheroidized particles, banded protrusions, pits, scratches, and local defects. The elemental composition of the specimen surface was detected using an energy dispersive spectrometer (EDS) attached to the SEM to analyze the changes in surface oxygen content and the distribution of major alloying elements before and after polishing. The three-dimensional surface topography, two-dimensional profile, and Rz and Ra roughness were measured using a laser confocal microscope to obtain the peak-to-valley undulations, local height differences, and typical profile curves of the surface, as well as to assist in verifying the roughness tester results. In this work, the Ra values reported in the text and figures are based on the TR200 results, while the Rz values and the three-dimensional topography are obtained from the laser confocal microscope.

Notably, during Q-DECMP, the continuous rotation of the anode and cathode keeps the workpiece and abrasives in continuous motion. The liquid bridges carried by the abrasives repeatedly form and rupture, and the effective machining region of the workpiece changes constantly. In the voltage range of 150–250 V, the current is only on the order of 0.06–0.10 A, making it difficult to obtain a continuous and stable current curve. Therefore, the current is reported as a range in the subsequent experimental parameters.

For the other quantitative results, three repeated experiments were conducted for each set of process parameters, except for the current, which is reported as a range as explained above. Ra and Rz were measured at five different positions on the cylindrical sidewall of each specimen, and the average value was used (*n* = 5). The line Ra values in the roughness profiles are the average of these five measurements. The final Ra and Rz values for each parameter set are the average of three repeated experiments (*n* = 3). MRR was calculated from the mass loss measured by a precision electronic balance (0.001 g) and is reported as the average of three repeated experiments (*n* = 3). EDS values are the average of three measurements at different points on each surface (*n* = 3). XPS spectra were obtained from representative specimens and are presented as single measurements. Error bars represent the standard deviation of repeated measurements.

## 4. Results and Discussion

### 4.1. Electrochemical Test Results

#### 4.1.1. EIS Analysis

The fitting results of the EIS data are shown in Figure 5a. At 0.01 Hz, the 1 mol/L NaCl-EG system exhibits the lowest impedance modulus of approximately 2.36 × 10^4^ Ω·cm^2^, while those of the 1 mol/L NaCl-H_2_O and 0.5 mol/L NaCl-EG systems are about 3.58 × 10^4^ and 2.50 × 10^5^ Ω·cm^2^, respectively. The low-frequency impedance modulus reflects the overall hindrance effect of the electrode interface. Therefore, the above results indicate that the interfacial inhibition effect on the TC4 surface is the strongest in 0.5 mol/L NaCl-EG. In contrast, the 1 mol/L NaCl-EG system has the lowest low-frequency impedance, suggesting that increasing the NaCl concentration facilitates charge transfer and correspondingly enhances the anodic reaction activity [18].

The phase angle curves further show that the 1 mol/L NaCl-EG system exhibits a broad high-phase-angle plateau in the medium-to-low frequency region, indicating a more pronounced capacitive character in its interfacial response. However, a broader plateau does not necessarily correspond to higher film protection, as the low-frequency impedance of this system remains the lowest among the three. The 0.5 mol/L NaCl-EG system displays a higher low-frequency impedance, indicating stronger barrier effects from its surface film or interfacial reaction. The impedance level of the 1 mol/L NaCl-H_2_O system falls between those of the two EG-based systems, but its phase angle varies more rapidly with frequency, indicating relatively less stable interfacial processes [19].

#### 4.1.2. Potentiodynamic Polarization Behavior

To investigate the potentiodynamic polarization behavior, polarization tests were conducted on the three solutions, and the resulting polarization curves are shown in Figure 5b. It can be observed that:

For the 0.5 mol/L NaCl-EG curve, the corrosion potential is approximately −0.65875 V. Upon entering the anodic region, the current rapidly transitions to a relatively low and stable plateau. Within the range of 0 to 0.5 V, the geometric mean of the current density is approximately 1.35 × 10^−6^ A/cm^2^, and the variation in log_10_|j| is only 0.072 decade. This result is consistent with its highest low-frequency impedance, indicating that a stable and strongly barrier-type passive state can be established in low-concentration NaCl-EG [20]. This system exhibits the best corrosion resistance; however, the stronger passivation also hinders the oxidation reaction of the Q-DECMP anode body, which is detrimental to improving material removal efficiency.

For the 1 mol/L NaCl-H_2_O curve, the corrosion potential is approximately −0.69075 V. During the subsequent anodic scan, the curve exhibits multiple current peaks and valleys. Among them, the current valley near −0.19 V corresponds to a state transition during the anodic reaction process and cannot replace the first valley as the corrosion potential [21]. In this system, within the range of −0.6 V to 0.2 V, the current density does not increase but rather decreases slightly, indicating the formation of a stable passivation region. Above 0.2 V, the current density increases significantly, entering the transpassive region, and pitting corrosion begins to occur on the workpiece surface.

For the 1 mol/L NaCl-EG curve, the corrosion potential is approximately −0.70675 V. After crossing the corrosion potential, the anodic current is significantly higher than that for 0.5 mol/L NaCl-EG. Subsequently, the overall current decreases with increasing potential and exhibits limited fluctuations over a wide potential range, displaying quasi-passive or incomplete passivation behavior. The surface reaction layer of this system can provide some suppression of the anodic process, but its barrier effect is weaker than that of 0.5 mol/L NaCl-EG. Therefore, the electrochemical results indicate that the glycol-based solution exhibits lower pitting susceptibility than the aqueous system, which is beneficial for surface smoothing.

The corrosion potential *E*_corr_ reflects the thermodynamic tendency for anodic dissolution of TC4 titanium alloy in different electrolyte systems. A negative shift in *E*_corr_ signifies an increased thermodynamic propensity for anodic oxidation. In contrast, the corrosion current *I*_corr_ characterizes the kinetic rate of the anodic dissolution process and serves as a critical parameter for evaluating the material removal capability during quasi-dry electrochemical mechanical composite polishing. A higher *I*_corr_ corresponds to a stronger anodic dissolution effect, which facilitates the material removal from the workpiece surface.

As presented in Table 4, the 1 mol/L NaCl-H_2_O system exhibits a more positive *E*_corr_ compared to the 1 mol/L NaCl-EG system; however, it demonstrates the highest *I*_corr_ among the three groups. This finding indicates superior charge transport capability within this electrolyte system, and the substantial corrosion current implies that it possesses the highest kinetic rate of anodic dissolution. Conversely, the 0.5 mol/L NaCl-EG system displays the most negative *E*_corr_, suggesting a strong thermodynamic tendency for anodic oxidation. Nevertheless, it exhibits the lowest *I*_corr_, thereby resulting in the weakest anodic dissolution effect.

**Figure 6:**
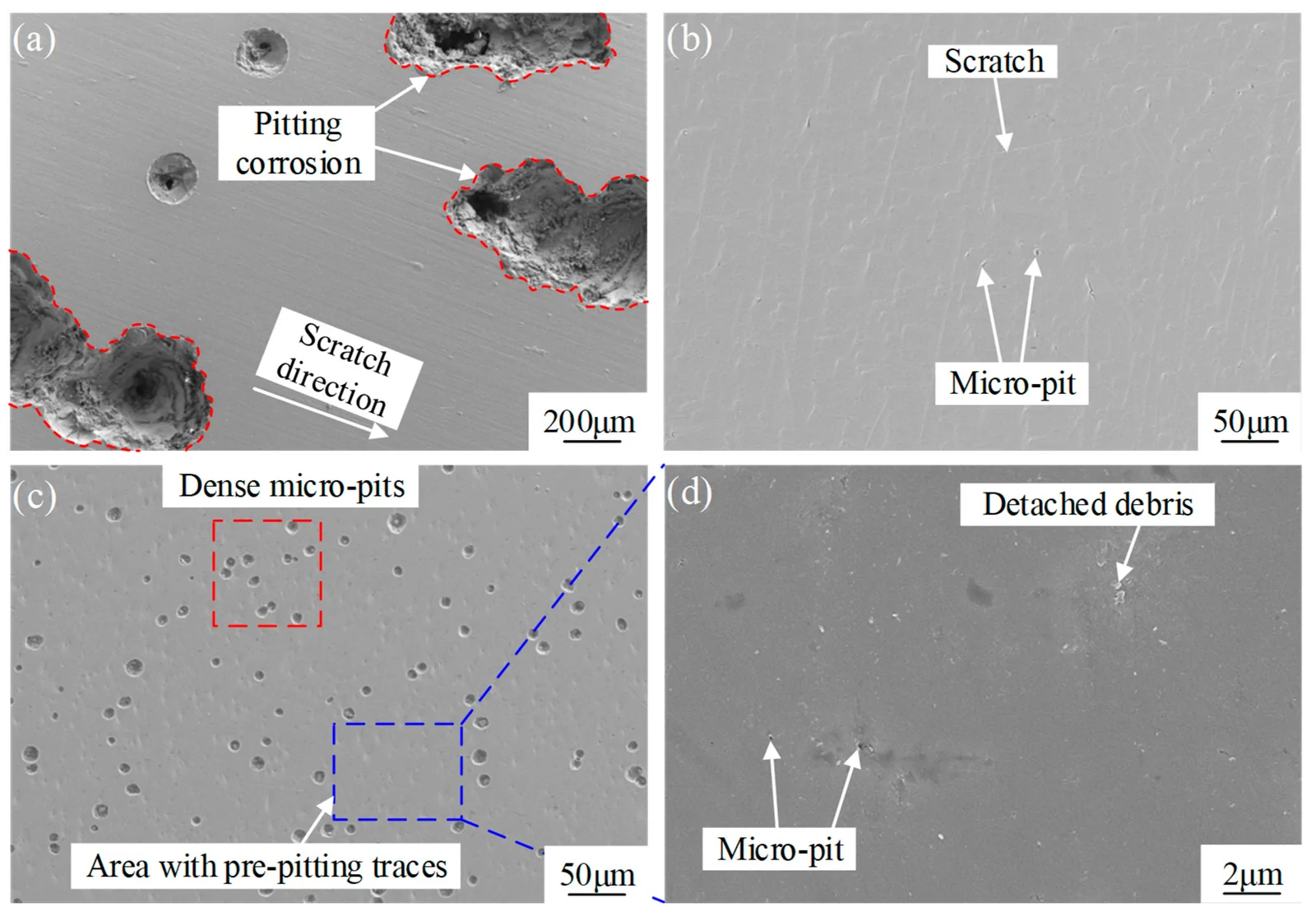
SEM surface morphologies of the workpiece after electrochemical testing: (**a**) SEM morphology of TC4 titanium alloy after polarization in 1 mol/L NaCl-H_2_O; (**b**) test result in 0.5 mol/L NaCl-EG system; (**c**) test morphology in 1 mol/L NaCl-EG system; (**d**) 5000× magnified morphology of the pit-free region in (**c**).

After electrochemical testing, the workpieces were examined using a ZEISS Sigma 360 SEM (Germany). The results are shown in Figure 6. After electrochemical testing in the 1 mol/L NaCl-H_2_O electrolyte, large surface pits can be observed at 50× magnification. From a macroscopic perspective, surface pits are clearly visible, along with distinct grinding marks, as shown in Figure 6a. Compared with Figure 6b, for the 0.5 mol/L NaCl-EG electrolyte, no pits are observed on the surface at 200× magnification. However, there are signs of pit initiation, and the grinding marks are not evident. This may be due to the formation of a passive film on the surface; the thick passive layer prevents pitting occurrence and also weakens the visibility of grinding marks. In contrast, compared to the workpiece tested in the 1 mol/L NaCl-H_2_O electrolyte, the workpiece surface tested in the 1 mol/L NaCl-EG electrolyte examined by SEM at 200× magnification only exhibits sparse and shallow pitting corrosion, as shown in Figure 6c. When further magnified to 5000× (as shown in Figure 6d), micro-pits and non-detached debris can be observed on the surface, with no grinding marks. This is because, under the 1 mol/L NaCl-EG electrolyte environment, the TC4 electrochemical test involves a higher NaCl concentration than 0.5 mol/L NaCl-EG. A higher concentration of Cl^−^ ions more readily penetrates the passive film and induces pitting initiation. Unlike the purely aqueous solution system, the glycol-based medium leads to much shallower pits, suggesting that the propagation of pitting corrosion in the depth direction is suppressed. However, the detailed mechanism still requires further verification. Consequently, only shallow micro-pits can form. This indicates that solvent polarity, viscosity, and proton activity are key factors determining the pitting morphology of TC4 [22].

**Figure 7:**
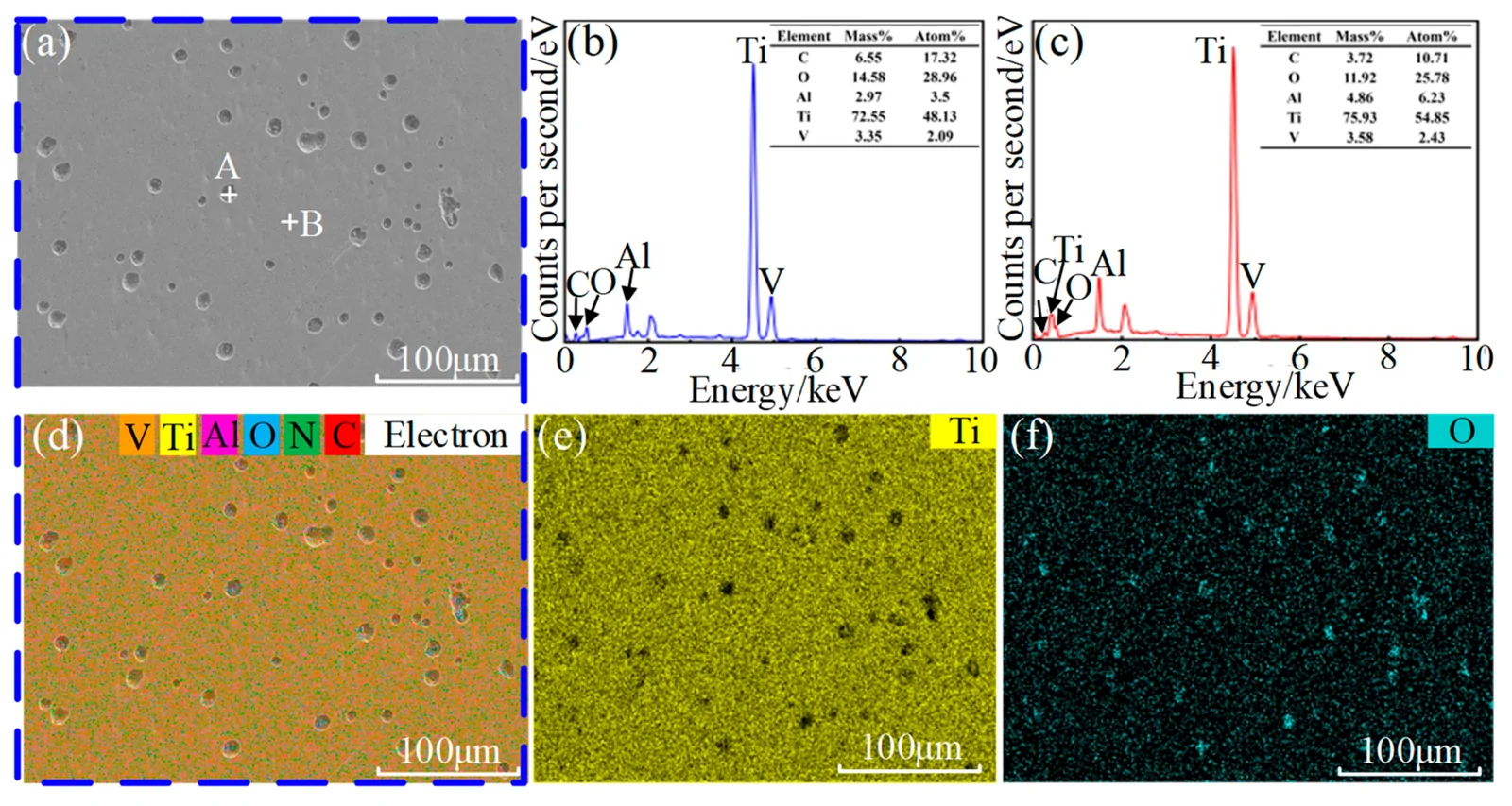
EDS mapping analysis of the surface after electrochemical testing: (**a**) Surface morphology of the workpiece after electrochemical testing; (**b**) EDS spectrum at point A; (**c**) EDS spectrum at point B; (**d**) overall EDS mapping distribution of major elements; (**e**) surface distribution of Ti element; (**f**) surface distribution of O element.

EDS mapping analysis was performed on the pitting corrosion pits formed after electrochemical testing in the 1 mol/L NaCl-EG electrolyte. The results are shown in Figure 7. On the non-pitted TC4 surface, the elemental distribution is uniform, primarily consisting of Ti, V, Al, C, and O. Compared with the initial elemental composition of the TC4 surface (as shown in Figure 2b), the Ti/O ratio decreases within the pitting pits, the C content increases, and the Al and V contents decrease. Based on the individual element mapping results, it is clearly observed that the pitting region exhibits a relatively higher O signal and a lower Ti signal, suggesting that the degree of surface oxidation or the distribution of oxygen-containing reaction products may differ from that of the flat areas. The enhanced O signal and weakened Ti signal in the pitting region indicate an increase in the thickness of the surface oxide layer or an enrichment of reaction products [23].

Under low-potential conditions, by integrating the EIS data, polarization curves, and post-polarization surface morphology, 0.5 mol/L NaCl-EG exhibits the highest interfacial impedance and a more stable low-current passive state. However, due to its lower NaCl concentration, the electrolytic conductivity is poorer than that of 1 mol/L NaCl-EG. The 1 mol/L NaCl-H_2_O system does not form a stable protective passive state and is accompanied by significant, large-scale localized corrosion. The 1 mol/L NaCl-EG system has the lowest interfacial impedance and relatively high anodic reaction activity. Its surface reaction displays quasi-passive characteristics, and the degree of pitting corrosion is significantly lower than that of the aqueous-based system. Considering the characteristics of the Q-DECMP, the workpiece surface is not in an immersed state. Under these conditions, compared to aqueous solutions, the higher-viscosity glycol is more conducive to the adhesion of the liquid film on abrasive grains. The higher proportion of NaCl provides higher electrical conductivity, which can accelerate the reaction rate. EDS results indicate that the surface chemical state changes after Q-DECMP, suggesting that the surface oxide/reaction layer is modified during processing. It should be noted that the bulk electrochemical results provide a basis for electrolyte selection, but the local interfacial conditions in Q-DECMP, which involve thin liquid films and liquid bridges, are not fully equivalent to those in bulk electrolyte tests [24]. In addition, EIS was not performed under the quasi-dry contact condition because, at a small EIS perturbation voltage, no effective electrochemical reaction occurs in the quasi-dry state, where a stable conductive path can only be established under a sufficiently high applied voltage [25]. Considering all these factors, the 1 mol/L NaCl-EG system was selected for subsequent polishing experiments.

### 4.2. Effect of Machining Voltage on Surface Quality

**Figure 8:**
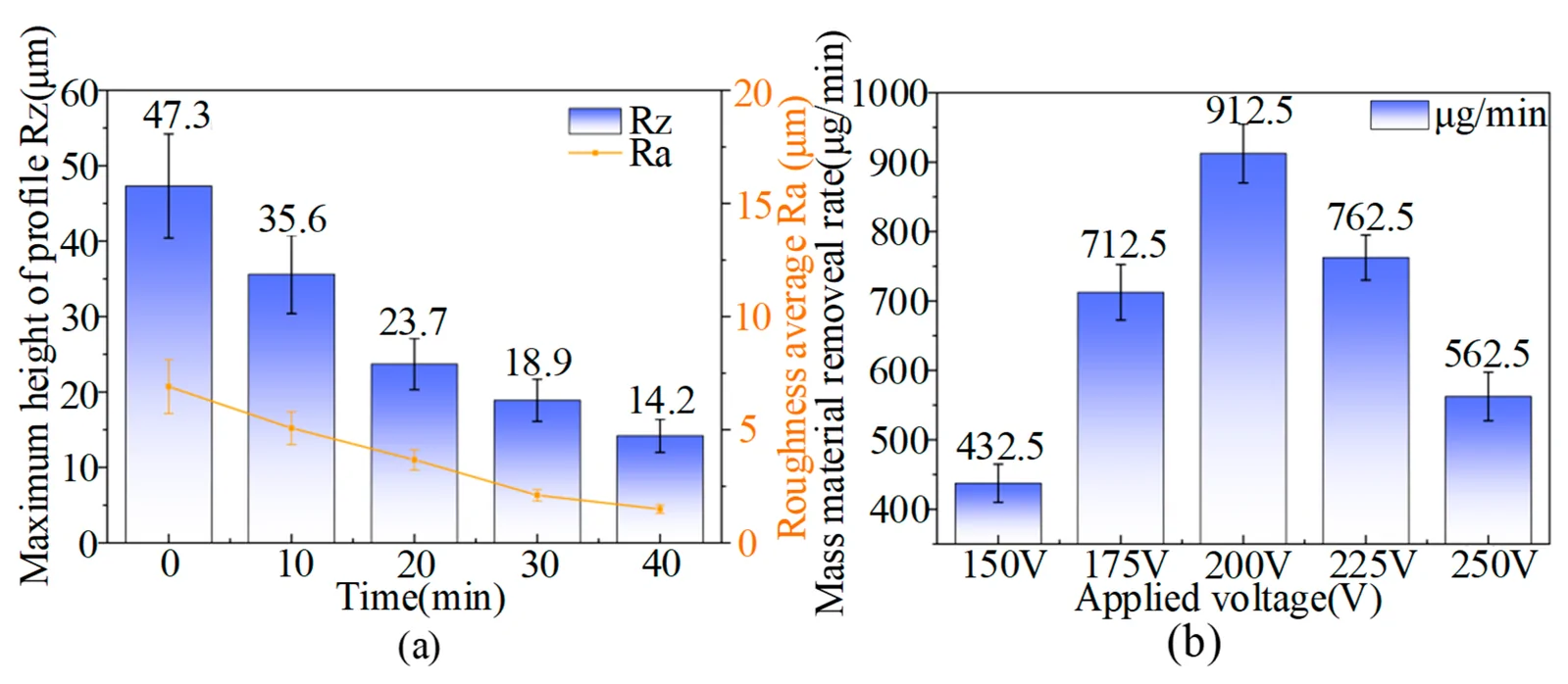
Material removal amount under different voltage parameters and surface roughness variation over different time intervals: (**a**) Surface roughness variation every 10 min at 200 V; (**b**) material removal amount for voltages ranging from 150 V to 250 V. The Ra and Rz values are presented as mean ± standard deviation (*n* = 3).

In this section, the machining voltage was varied from 150 V to 250 V, while the electrolyte-to-abrasive mass ratio and the linear velocity were kept constant at 1:20 and 2.0 m/s, respectively, and its effects on material removal and surface leveling in Q-DECMP were investigated. Figure 8a further shows the change in surface roughness over machining time under 200 V. The initial SLM-TC4 titanium alloy surface exhibited distinct peak-to-valley undulations, with an Rz of 47.3 μm and an Ra of 6.89 μm. After 10 min of machining, Rz rapidly decreased to 35.6 ± 5.2 μm, indicating that highly protruding surface defects were quickly reduced through the combined action of localized anodic dissolution and the mechanical action of the abrasive grains. As the machining time extended to 20 and 30 min, Rz further decreased to 23.7 ± 3.4 μm and 18.9 ± 2.8 μm (*n* = 3), respectively, demonstrating a continuous reduction in the peak-to-valley height difference. After 40 min of machining, Rz decreased to 14.2 ± 2.2 μm, and Ra decreased to 1.49 ± 0.20 μm, representing approximately a 78.4% reduction from the initial Ra.

Figure 8b presents the material removal results after 40 min of machining at various voltages. To more intuitively evaluate the material removal capability, the mass removed per minute was used as the material removal metric. As shown in Figure 8b, the average MRR first increased and then decreased as the voltage increased. When the voltage increased from 150 V to 200 V, the average MRR increased from approximately 432.5 ± 27.5 μg/min to 912.5 ± 42.5 μg/min, indicating that a higher voltage enhances the localized anodic dissolution in the contact area of the abrasive liquid film, thereby improving the material removal efficiency at the peak regions. In this range, the increased voltage raises the local current density at the abrasive–workpiece contact regions, promotes the breakdown of the surface oxide film, and accelerates the anodic dissolution of the peak material.

When the voltage was further increased to 225 V and 250 V, the average MRR decreased to approximately 762.5 ± 32.5 and 562.5 ± 35 μg/min, respectively. This decrease can be attributed to the excessively high local current density at high voltages. On the one hand, the localized Joule heating may cause partial decomposition of the ethylene glycol-based electrolyte and reduce the stability of the thin liquid film and liquid bridges, leading to more frequent rupture of the liquid bridges and a shorter effective conduction time. On the other hand, the excessively high local current density intensifies pitting corrosion, so that part of the current is consumed by pitting rather than by effective leveling dissolution, resulting in a lower current efficiency [26]. These two effects together lead to the decrease in MRR at voltages above 200 V.

Considering both the MRR and the change in roughness, it can be seen that Q-DECMP under 200 V not only provides high material removal efficiency but also continuously reduces the peak-to-valley undulations on the SLM-TC4 titanium alloy surface. This suggests that at this voltage, the conductive liquid film on the abrasive surface can effectively establish localized current channels at the surface peak regions, promoting a preferential anodic dissolution of the peaks [27]. Simultaneously, the mechanical action of the abrasives assists in removing reaction products, allowing the surface high peaks to be progressively reduced. Therefore, a combination of high removal efficiency and good surface leveling effect can be achieved under the 200 V condition.

**Figure 9:**
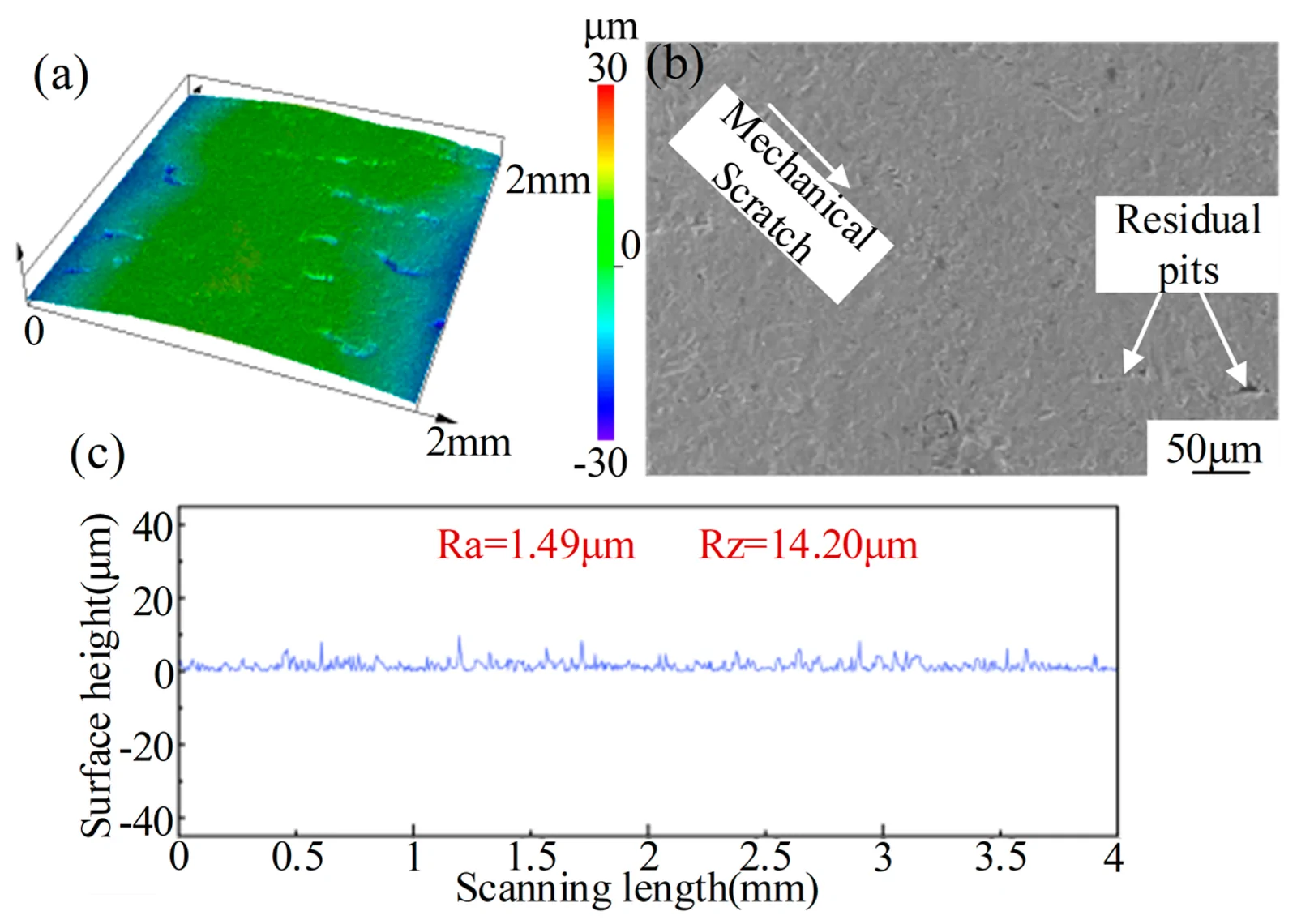
Surface morphology and roughness curves after 40 min processing with near-optimal voltage parameters of 200 V. (**a**) Laser confocal microscopy in a 2 × 2 mm area. (**b**) SEM surface morphology. (**c**) Ra roughness curve under average roughness. The Ra and Rz values are presented as mean ± standard deviation (*n* = 3).

Laser confocal microscopy and scanning electron microscopy (SEM) were used to characterize the surface morphology of the workpiece machined under the 200 V condition, and the results are shown in Figure 9 (scan area: 2 mm × 2 mm). As seen in Figure 9a, besides some residual deep pits, the surface also exhibited scratches that affected the surface quality (Figure 9b). This indicates that during machining, the mechanical action was stronger than the electrochemical action, and the appearance of scratches further deteriorated the surface morphology. Although the MRR is high under this condition, the remaining crevices on the surface limit the reduction in roughness, with Ra reaching 1.49 μm and Rz reaching 14.2 ± 2.2 μm (Figure 9c). The strong mechanical action, while improving the MRR and accelerating the renewal of surface products, also leads to the formation of significant irregular scratches. Therefore, appropriately reducing the surface linear velocity may be more conducive to obtaining good surface quality.

### 4.3. Effect of Electrolyte Formulation on Surface Quality

In Q-DECMP, the material removal amount is jointly determined by the applied voltage and the electrolyte conductivity. Under the conditions of a fixed machining voltage of 200 V and a fixed linear velocity of 1.0 m/s, the electrolyte-to-abrasive mass ratio was varied among 1:10, 1:20, and 3:40 to study its effect on surface quality. When the voltage is fixed at 200 V, the conductivity of the NaCl-EG electrolyte depends on the NaCl concentration, while the width of the conductive channel is determined by the electrolyte supply rate. When 0.5 mol/L NaCl-EG solution is used, its ion concentration is lower than that of the 1 mol/L solution, resulting in lower conductivity; although this concentration is conducive to forming a passivation film and effectively suppressing over-corrosion, under the high-voltage condition of 200 V, the current density is too low to achieve the ideal polishing results. As the NaCl concentration increases, the electrolyte conductivity gradually rises, and the number of Cl^−^ ions also increases, thereby enhancing the pitting effect. Increasing the electrolyte ratio leads to a larger amount of NaCl, which causes an increase in the size and depth of pitting pits on the workpiece surface, subsequently deteriorating the surface flatness of the TC4 substrate [28].

**Figure 10:**
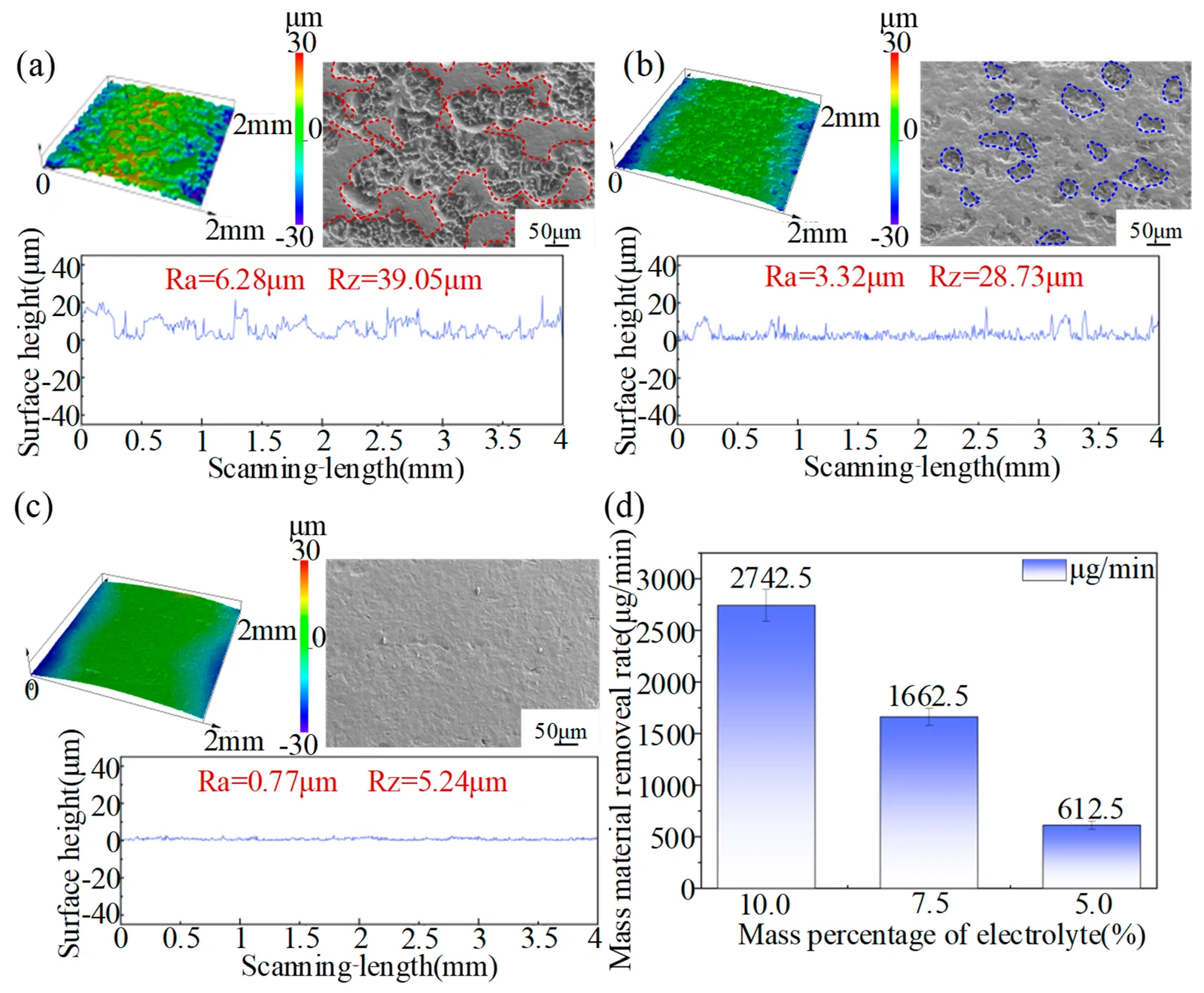
Surface morphology of materials under different electrolyte ratios: (**a**) Electrolyte-to-abrasive mass ratio of 1:10; (**b**) electrolyte-to-abrasive mass ratio of 3:40; (**c**) electrolyte-to-abrasive mass ratio of 1:20; (**d**) MRR under different electrolyte ratios. The Ra and Rz values are presented as mean ± standard deviation (*n* = 3).

Using an electrolyte-to-abrasive mass ratio of 1:10 (only varying the ratio of electrolyte to abrasives), the current during processing at a voltage of 200 V is 0.18–0.22 A. The higher electrolyte content allows more electrolytes to accumulate at the bottom of the abrasives. At this point, the conductive channel no longer relies solely on the liquid film and liquid bridge; there also exists a direct conduction path through the electrolyte that submerges the abrasives. The increased current significantly enhances the electrolytic action. Although the MRR is as high as 2742.5 ± 154 μg/min (*n* = 3, as shown in Figure 10d), the pitting pits are noticeably enlarged (Figure 10a). It can be observed from the figure that areas not completely pitted still exist on the side surface of the workpiece, but the pitting that has already occurred leads to deteriorated surface roughness, with an Ra of 6.28 ± 1.5 μm, only slightly more favorable than the unprocessed surface. To improve the surface quality of the workpiece under this ratio, lower voltage and shorter processing time should be considered.

The processing result using an electrolyte-to-abrasive mass ratio of 3:40 is shown in Figure 10b. Under a voltage of 200 V, the initial current is 0.12–0.18 A. After 40 min of processing, the surface roughness reaches Ra 3.32 ± 0.7 μm, and the MRR is 1662.5 ± 85 μg/min (*n* = 3). After processing, the surface is covered with pitting micro-pits, and the surface quality is significantly inferior to the result under the near-optimal process conditions, as shown in Figure 10c. With this ratio, the higher electrolyte amount leads to a higher initial current, and a large number of pitting pits appear on the processed surface, severely degrading the surface quality. It is worth noting that under this ratio, the electrolyte accumulates at the bottom of the polishing tank, creating a processing environment slightly better than the abrasive surface adhesion state where conduction relies solely on liquid bridges. This may cause the electrolytic action to be slightly stronger than the mechanical removal action. Although the surface oxide layer is removed, the enhanced electrolytic action induces pitting, ultimately resulting in surface quality inferior to that achieved with a 1:20 electrolyte-to-abrasive mass ratio [29].

The results above reveal a clear trade-off between MRR and surface quality at different electrolyte-to-abrasive mass ratios. At a ratio of 1:10, the electrolyte content is the highest, and the conductive path no longer relies solely on the liquid film and liquid bridges; instead, a direct conduction path through the electrolyte that submerges the abrasives is established. As a result, the current increases to 0.18–0.22 A, and the MRR reaches 2742.5 μg/min. However, the increased current also intensifies the electrolytic action, leading to enlarged pitting pits and a surface roughness of Ra 6.28 μm, which is only slightly better than the unprocessed surface. At a ratio of 3:40, the MRR decreases to 1662.5 μg/min, and the surface roughness improves to Ra 3.32 μm, but pitting micro-pits are still evident. At a ratio of 1:20, the MRR further decreases to 612.5 μg/min, but the surface roughness reaches the best value of Ra 0.77 μm. These results indicate that increasing the electrolyte content enhances the MRR but simultaneously promotes pitting and surface deterioration, whereas a moderate electrolyte content provides a better balance between removal efficiency and surface quality. Since the optimization criterion of this work is surface roughness, the 1:20 ratio was selected as the near-optimal condition. A multi-response optimization considering both MRR and surface roughness will be carried out in future work.

### 4.4. Effect of the Relative Speed Between Abrasives and Workpiece on Surface Quality

**Figure 11:**
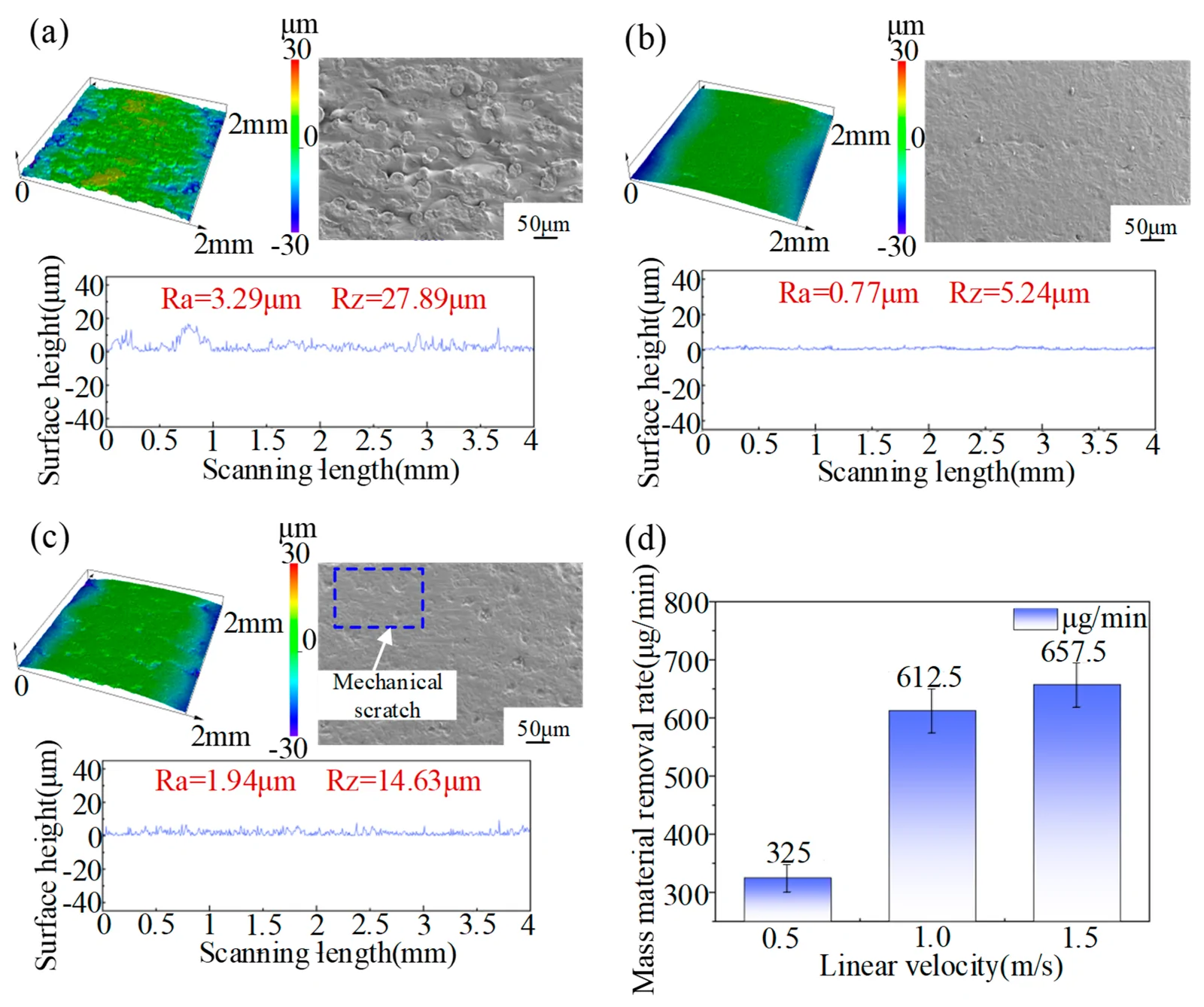
Surface morphology of the material at different linear velocities: (**a**) Surface morphology at a linear velocity of 0.5 m/s; (**b**) surface morphology at a linear velocity of 1 m/s; (**c**) surface morphology at a linear velocity of 1.5 m/s; (**d**) bar chart of material removal amount at the three linear velocities. The Ra and Rz values are presented as mean ± standard deviation (*n* = 3).

Higher relative speeds between abrasives and workpiece enhance mechanical action, facilitating the faster removal of surface passivation films. However, excessively strong mechanical action may cause surface scratches, degrading surface quality. Theoretically, optimal polishing performance is achieved when mechanical and electrochemical actions reach a dynamic, controllable, and stable equilibrium, with a steady current density serving as key evidence of this balance. In addition, high speeds generate greater heat accumulation, which may induce thermal decomposition of ethylene glycol, thereby affecting the polishing results. To investigate the effect of linear velocity on the surface polishing of TC4 titanium alloy, the workpiece sidewall linear velocity was varied among 0.5, 1.0, and 1.5 m/s, while the machining voltage and the electrolyte-to-abrasive mass ratio were kept constant at 200 V and 1:20, respectively. The results are shown in Figure 11.

Under conditions where only the linear velocity was varied, processing was first performed at 0.5 m/s for 40 min. As shown in Figure 11a, residual unremoved spheroidized particles remained on the workpiece surface. The MRR was 325 ± 24 μg/min. The low MRR indicated an inability to effectively eliminate surface defects. At a low linear velocity, mechanical action was weak, making it difficult to rapidly remove the naturally formed surface oxide film. Although no obvious scratches were observed, the surface roughness was as high as Ra 3.29 ± 0.7 μm, and banded protrusions and spheroidized particles severely degraded the surface quality.

When the linear velocity was increased to 1.0 m/s, the results are shown in Figure 11b. SEM images at 200× magnification revealed no remaining unmachined areas on the workpiece surface, with defects almost entirely removed except for some deep crevice defects that were not fully eliminated. The MRR at over 40 min of processing was 612.5 μg/min, approximately 1.9 times that at 0.5 m/s. The surface roughness decreased to Ra 0.77 ± 0.16 μm, a reduction of about 88.8% compared to the initial surface (Ra 6.89 μm), and the surface quality was significantly better than that obtained at 0.5 m/s.

When the linear velocity was further increased to 1.5 m/s, the higher linear velocity enabled more efficient removal of the natural oxide film, extending the duration of electrolytic action on the substrate. As shown in the SEM image in Figure 11c, banded protrusions were completely removed, but scratches and micropits resembling pitting corrosion appeared on the surface, leading to degraded surface quality. Under these conditions, the surface roughness was Ra 1.94 ± 0.32 μm, higher than that at 1.0 m/s; the MRR was 657.5 ± 38.5 μg/min, also higher than that at 1.0 m/s. Although the original surface defects were completely eliminated, the resulting micropits and scratches deteriorated surface quality. At a high linear velocity, mechanical removal was enhanced, but if the duration of electrochemical dissolution was excessive, selective phase dissolution might occur, forming micropits.

Both excessively low and high linear velocities are detrimental to achieving the desired surface quality, with 1.0 m/s being the optimal parameter within the experimental range. At a linear velocity of 0.5 m/s, mechanical action was too weak to effectively remove surface defects such as natural oxide films and spheroidized particles. The MRR was only 325 μg/min, and the surface roughness Ra reached as high as 3.29 μm. When the linear velocity was increased to 1.0 m/s, mechanical and electrolytic actions approached a balance, with original surface defects essentially removed. The MRR increased to 612.5 μg/min, and the roughness Ra significantly decreased to 0.77 μm, yielding the best surface quality. Upon further increasing the linear velocity to 1.5 m/s, although the MRR continued to rise to 657.5 μg/min, the excessively strong mechanical action induced surface scratches. Simultaneously, the prolonged electrolytic action led to selective phase dissolution, forming pitting micropits, which increased the surface roughness back to Ra 1.94 μm, degrading the surface quality instead. Although the MRR at 1.5 m/s was slightly higher than that at 1.0 m/s (657.5 vs. 612.5 μg/min, an increase of only about 7.3%), the surface roughness deteriorated significantly from Ra 0.77 to 1.94 μm. Since the optimization criterion of this work is surface roughness (Ra), 1.0 m/s is therefore superior to 1.5 m/s for achieving the desired surface quality. Therefore, only at a moderate linear velocity, where mechanical removal and electrochemical dissolution achieve dynamic equilibrium, can favorable polishing performance be obtained.

### 4.5. Comparative Experimental Analysis

**Figure 12:**
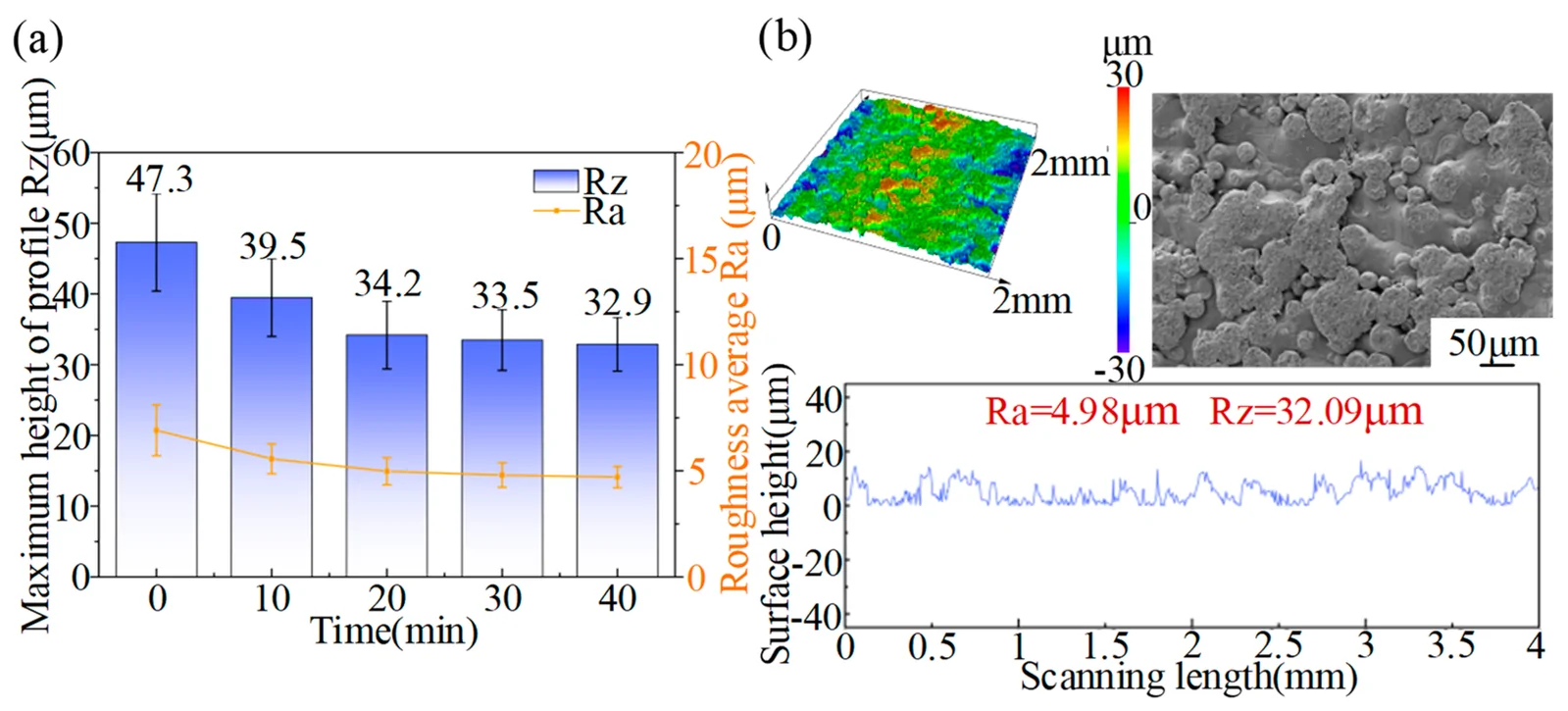
Surface morphology of the material after 40 min of pure mechanical polishing: (**a**) Ra and Rz evolution over polishing time (sampled every 10 min); (**b**) 3D topography, SEM image, and typical line roughness curve of the final surface. The Ra and Rz values are presented as mean ± standard deviation (*n* = 3).

After 40 min of purely mechanical machining (without voltage application), as shown in Figure 12a, the surface roughness Ra exhibits a gradual decreasing trend with prolonged polishing time, but the decreasing rate slows down significantly. After 20 min of polishing, the Ra values tend to level off, indicating that the polishing process has approached or reached the limit under the current conditions, with the final Ra stabilizing at approximately 4.98 ± 0.81 μm. The spheroidized particles and step effect on the TC4 titanium alloy surface were not effectively removed (as shown in the laser confocal and SEM 3D morphologies in Figure 12b), resulting in poor surface flatness with a roughness of Ra 4.98 μm. In contrast, after 40 min of Q-DECMP, as shown in Figure 11b, the spheroidized particles and step effect on the surface were completely removed, leaving only some deep crevice defects. The surface roughness significantly decreased from an initial Ra of 6.89 μm to Ra 0.77 μm. Purely mechanical action relies solely on the mechanical scratching of abrasive grains to remove material. Abrasive grains with a particle size much larger than the spheroidized particles primarily contact the surface peaks, resulting in limited mechanical action that can only remove small spheroidized particles. The MRR for purely mechanical machining is only 220 ± 30 μg/min (*n* = 3), and the cumulative removal mass within 40 min is merely 8.8 mg, indicating a very low MRR.

Pure mechanical polishing removes material solely through abrasive scratching. Since the abrasive particle size is much larger than that of the spheroidized particles, the abrasives mainly contact the surface peaks, resulting in limited mechanical action that can only remove small spheroidized particles. The material removal rate of pure mechanical polishing is only 220 μg/min, and the cumulative removed mass after 40 min is only 8.8 mg, indicating a very low removal efficiency.

To quantitatively evaluate the material removal capability of the two processing methods, the mass removal rate (MRR) was adopted as the metric:

$$MRR=\frac{∆m}{t}$$

where ∆m is the removed mass and t is the processing time.

The material removal rate of pure mechanical polishing was 220 μg/min. After 40 min of Q-DECMP, the spheroidized particles and the step effect on the surface were completely eliminated, and the roughness decreased from the initial Ra 6.89 μm to Ra 0.77 μm, corresponding to a material removal rate of 612.5 μg/min, approximately 2.78 times that of pure mechanical polishing. Based on the removed mass over 40 min, the mechanical contribution was about 8.8 mg, while the net increase attributed to the introduction of electrochemical dissolution was about 15.7 mg, corresponding to apparent contributions of approximately 36% and 64%, respectively. These results indicate that electrochemical dissolution plays a dominant role in the polishing process, while mechanical action serves an auxiliary role [30]. In Q-DECMP, electrochemical oxidation is coupled with mechanical removal, which enables the simultaneous removal of both spheroidized particles and the step effect, thereby significantly improving the material removal efficiency. It should be noted that these percentages are apparent contributions rather than strictly separated ones, since only Q-DECMP and pure mechanical polishing experiments were conducted in this work.

EDS analysis was performed on the unpolished surface, the Q-DECMP surface, and the purely mechanically polished surface. The average atomic percentage was determined at five randomly selected points on each surface, and the results are shown in Table 5. After purely mechanical polishing, the surface Ti/O atomic ratio increased, indicating that the mechanical action can effectively remove the surface oxide film. The Ti/O atomic ratio after Q-DECMP was also higher than that of the unpolished surface. However, due to different levels of surface carbon contamination under the two polishing methods, it is difficult to directly compare their effectiveness in removing the oxide film based solely on the Ti/O atomic ratio.

### 4.6. Response Surface Analysis of Voltage and Linear Speed on Ra

To further analyze the correlation between voltage and linear speed on surface roughness, a quadratic response surface model for Ra was established based on the above experimental results. The regression equation in coded form is:

$$Ra=0.8390-0.1504A-0.4505B-0.0457AB+0.3154A^{2}+1.0979B^{2}$$

where Ra is the surface roughness (μm); A and B are the coded values of voltage (V) and linear speed (m/s), respectively, ranging from −1 to 1; AB is the interaction term; and $A^{2}$ and $B^{2}$ are the quadratic terms. The coefficients can be directly compared to evaluate the relative effects of each term on Ra.

**Figure 13:**
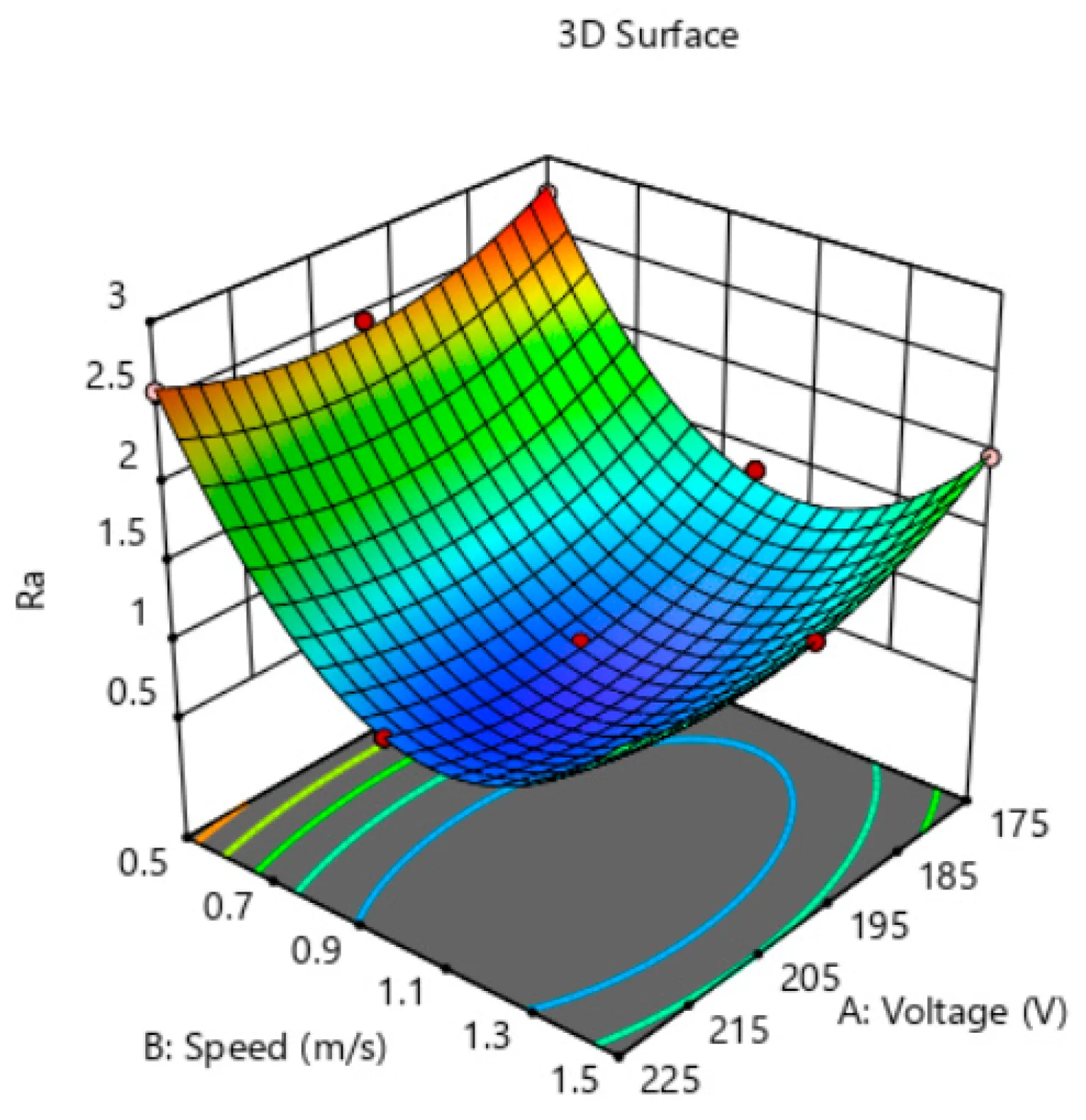
3D response surface of Ra as a function of voltage and linear speed.

The model *p*-value is <0.01 and R^2^ = 0.9967, indicating that the model can well describe the effects of voltage and linear speed on Ra. The absolute coefficient of the linear term for speed (0.4505) is larger than that for voltage (0.1504), suggesting that linear speed has a more significant effect on Ra within the experimental range. Both quadratic coefficients are positive, indicating an upward-opening response surface with a minimum. Although the interaction term AB is not statistically significant, its coefficient of −0.0457 still reflects a certain coupling trend between voltage and linear speed: voltage governs the degree of electrochemical oxidation, while linear speed affects the mechanical removal efficiency of the oxide layer and abrasives. A proper match between them is required to achieve a lower Ra. Combined with the 3D response surface in Figure 13, the minimum Ra appears around 195–205 V and 1.1–1.3 m/s, and Ra increases when deviating from this region, indicating an optimal matching range between voltage and linear speed. This inference agrees with the above experimental results and can serve as a reference for subsequent process parameter optimization.

### 4.7. Evolution of Surface Morphology During Q-DECMP

**Figure 14:**
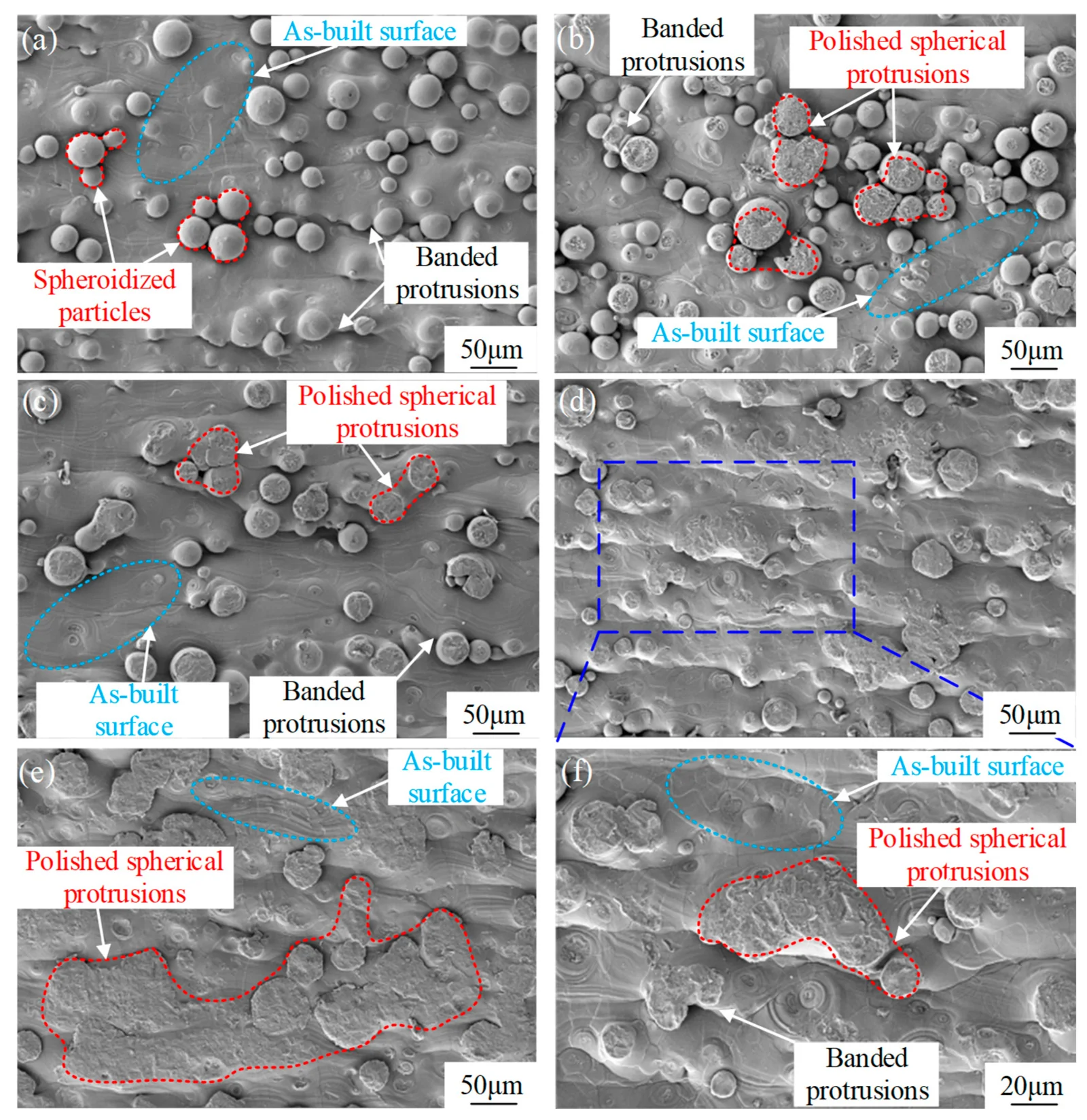
Evolution of the surface morphology of SLM-TC4 titanium alloy during Q-DECMP processing (**a**) Initial surface; (**b**) processing for 30 s; (**c**) processing for 60 s; (**d**) processing for 180 s; (**e**) processing for 360 s; (**f**) magnified view of (**d**).

To more clearly illustrate the processing principle, SEM inspection was performed on specimens with different processing times using a German ZEISS Sigma 360 system, with the results shown in Figure 14. Due to the inability of molten metal to spread into a flat track before solidification during the SLM process, it contracts into droplets under the action of surface tension, forming spheroidized particles. The molten metal fails to adequately fuse with the underlying layer, creating banded protrusions [31]. Figure 14a shows the surface morphology at 200× magnification in the as-built condition. To visualize the evolution of surface morphology during the Q-DECMP polishing process, SEM observations of surface morphology were conducted on TC4 circular workpieces at different processing times. After 30 s of processing, larger spheroidized particles were consumed, while small-sized spherical particles remained unprocessed due to lack of contact with abrasive grains. Additionally, because the processing time was too short and the surface removal amount was small, the banded protrusions and the original surface remained unchanged, as shown in Figure 14b.

When the processing time was extended to 60 s, small spheroidized particles were also removed, and large spheroidized particles were almost completely eliminated. Due to the relatively small amount of material removed, the banded protrusions remained unchanged, and the original surface was still unprocessed, as shown in Figure 14c. Continuing to extend the processing time to 180 s, it can be clearly observed that raised spheroidized particles above the average surface were completely removed, while small spheroidized particles below the average surface were still not fully cleaned. The raised banded protrusions were subjected to processing, but the overall material removal amount on the surface was still not large, and the original surface matrix remained unprocessed, as shown in Figure 14d,f, where Figure 14f is a 400× magnification view. When the processing time was extended to 360 s, the spheroidized particles were completely removed, the banded protrusions were significantly reduced, the original matrix surface previously covered by defects gradually became exposed, and the matrix itself showed no obvious excessive dissolution, as shown in Figure 14e. This indicates that the synergistic effect of abrasive scratching and electrolytic action achieves localized removal of surface protrusions without damaging the recessed areas [32].

**Figure 15:**
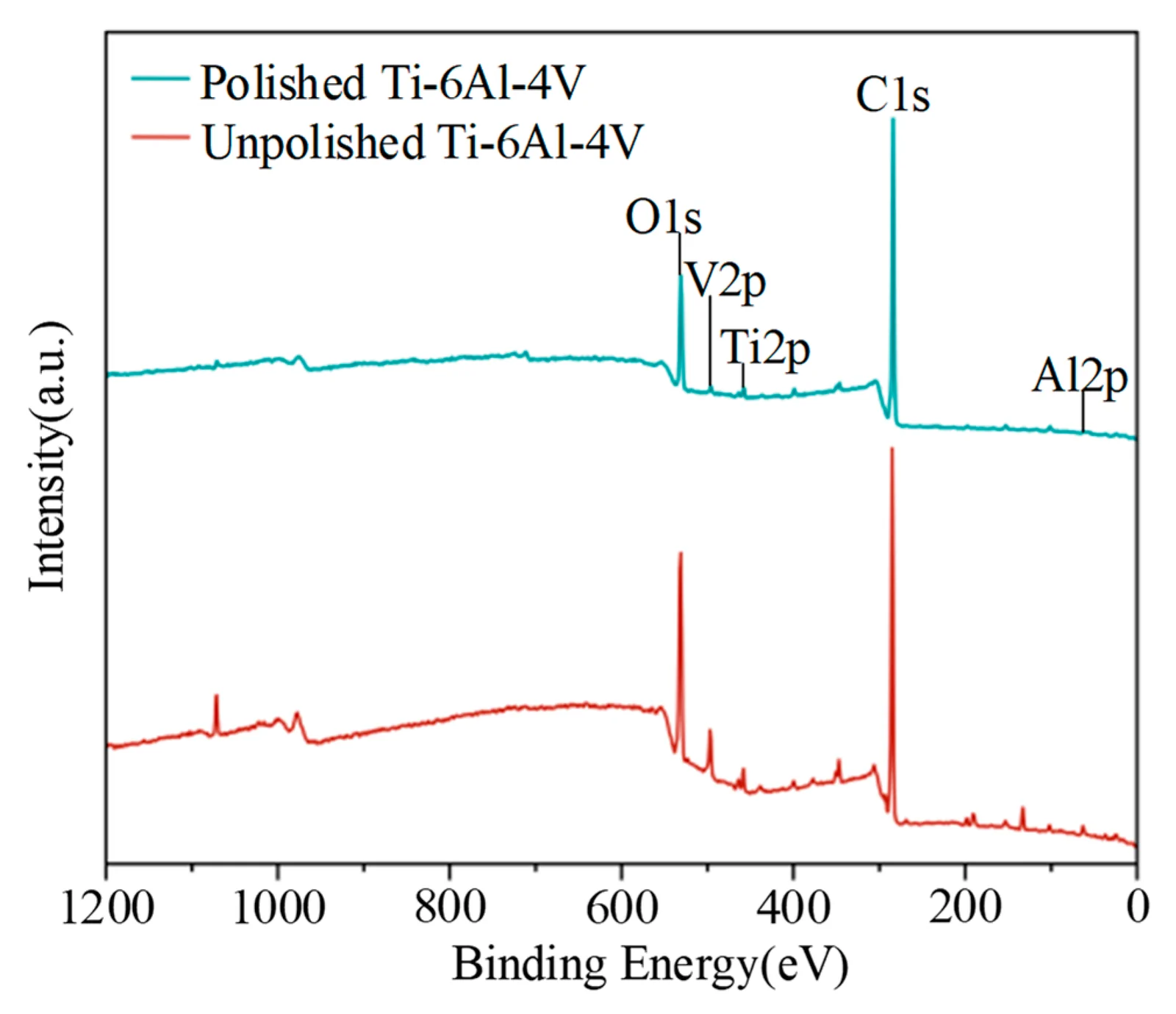
XPS full spectrum analysis before and after TC4 polishing.

**Figure 16:**
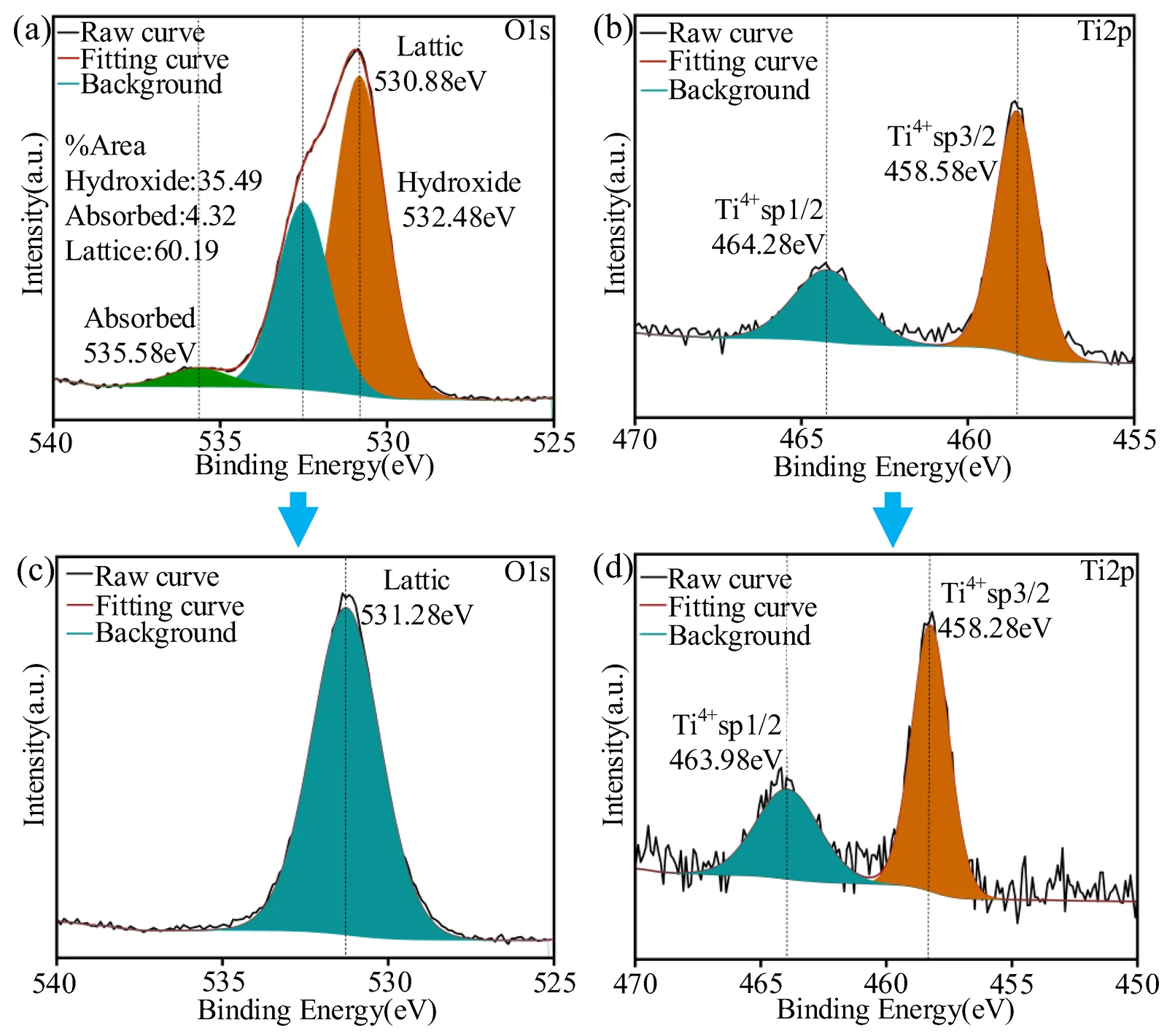
XPS fitting curves of the workpiece surface before and after polishing: (**a**) O 1s fitting curve before polishing; (**b**) Ti 2p fitting curve before polishing; (**c**) O 1s fitting curve after polishing; (**d**) Ti 2p fitting curve after polishing.

To analyze the changes in elemental composition and chemical state on the surface of TC4 titanium alloy before and after Q-DECMP, XPS testing was performed on the unpolished and polished specimens. All high-resolution spectra were charge-corrected using the C 1s peak. The XPS survey spectra results are shown in Figure 15, and the O 1s and Ti 2p high-resolution spectra along with their fitting results are shown in Figure 16. The survey spectra analysis in Figure 15 indicates that C 1s, O 1s, Ti 2p, V 2p, and Al 2p signals were detected on both the unpolished and polished surfaces, with no new distinguishable elemental peaks appearing after polishing, suggesting that the surface elemental species detectable by XPS did not change before and after Q-DECMP treatment [33].

Figure 16a,b show the O 1s and Ti 2p high-resolution spectra of the specimen before polishing. The O 1s spectrum of the unpolished surface exhibits a distinctly asymmetric peak shape, which can be fitted into three components: metal oxide oxygen at 530.88 eV (peak area percentage 60.19%), hydroxyl oxygen at 532.48 eV (35.49%), and surface adsorbed water oxygen at 535.58 eV (4.32%). In the Ti 2p spectrum, the Ti 2p_3_/_2_ and Ti 2p_1_/_2_ peaks are located at 458.58 eV and 464.28 eV, respectively, with a spin-orbit splitting energy of 5.70 eV, corresponding to the Ti^4+^ oxidation state, indicating that the unpolished surface is primarily composed of TiO_2_-type oxides [34].

Figure 16c,d show the O 1s and Ti 2p high-resolution spectra of the specimen after polishing. After polishing, the O 1s spectrum exhibits a symmetric single peak, with the peak center located at 531.28 eV, shifted by 0.40 eV to a higher binding energy compared to the metal oxide oxygen peak (530.88 eV) of the unpolished specimen [35]. Under the current fitting conditions, no independent hydroxyl oxygen or adsorbed water oxygen shoulder peaks were resolved, indicating that surface hydroxylation and adsorbed species were significantly weakened or disappeared after Q-DECMP treatment [36]. In the Ti 2p spectrum after polishing, the Ti 2p_3_/_2_ and Ti 2p_1_/_2_ peaks are located at 458.28 eV and 463.98 eV, respectively, with the spin-orbit splitting energy remaining at 5.70 eV, indicating that the outermost surface still predominantly consists of the Ti^4+^ oxidation state. Compared to the unpolished condition, the entire Ti^4+^ doublet peaks shifted by 0.30 eV toward lower binding energy after polishing, while the spacing between the two peaks remained unchanged. This shift reflects changes in the local chemical environment and electronic structure of Ti^4+^ [37].

Based on the comprehensive analysis of the survey spectra, O 1s, and Ti 2p spectra, Q-DECMP treatment primarily changes the oxygen-containing species composition on the outermost surface of TC4, manifested as a significant weakening of hydroxylation and adsorbed components at high binding energies. The treated surface still predominantly features the Ti^4+^ oxidation state. This indicates that the combined polishing process can effectively remove or refresh the oxidation/adsorption reaction products on the original surface, but the sample will still generate a new Ti^4+^-containing surface oxide layer during processing and exposure to air [38]. This result is consistent with the dynamic mechanism of Q-DECMP, which involves “formation of a surface reaction layer-mechanical removal-reaction of the new surface.” As TC4 undergoes electrochemical oxidation as the anode and is simultaneously subjected to mechanical removal by abrasive grains, the surface chemical state undergoes continuous renewal, and the Ti^4+^ signals detected by XPS precisely reflect this dynamic process.

### 4.8. Flattening Mechanism

Based on the experimental results of process parameter optimization, observations of surface morphology evolution, and analysis of surface chemical states, a proposed efficient and localized planarization mechanism of Q-DECMP on SLM-TC4 titanium alloy is described below. The core of this proposed mechanism lies in the synergistic effect of “localized anodic dissolution” and “mechanical-assisted removal,” which is specifically manifested in the preferential contact of the conductive liquid bridge with surface peaks, the coupling of electrochemical dissolution and mechanical scraping at the peak regions, and ultimately the gradual reduction in surface asperities. It should be noted that this mechanism is proposed based on the present experimental evidence and has not yet been directly demonstrated.

**Figure 17:**
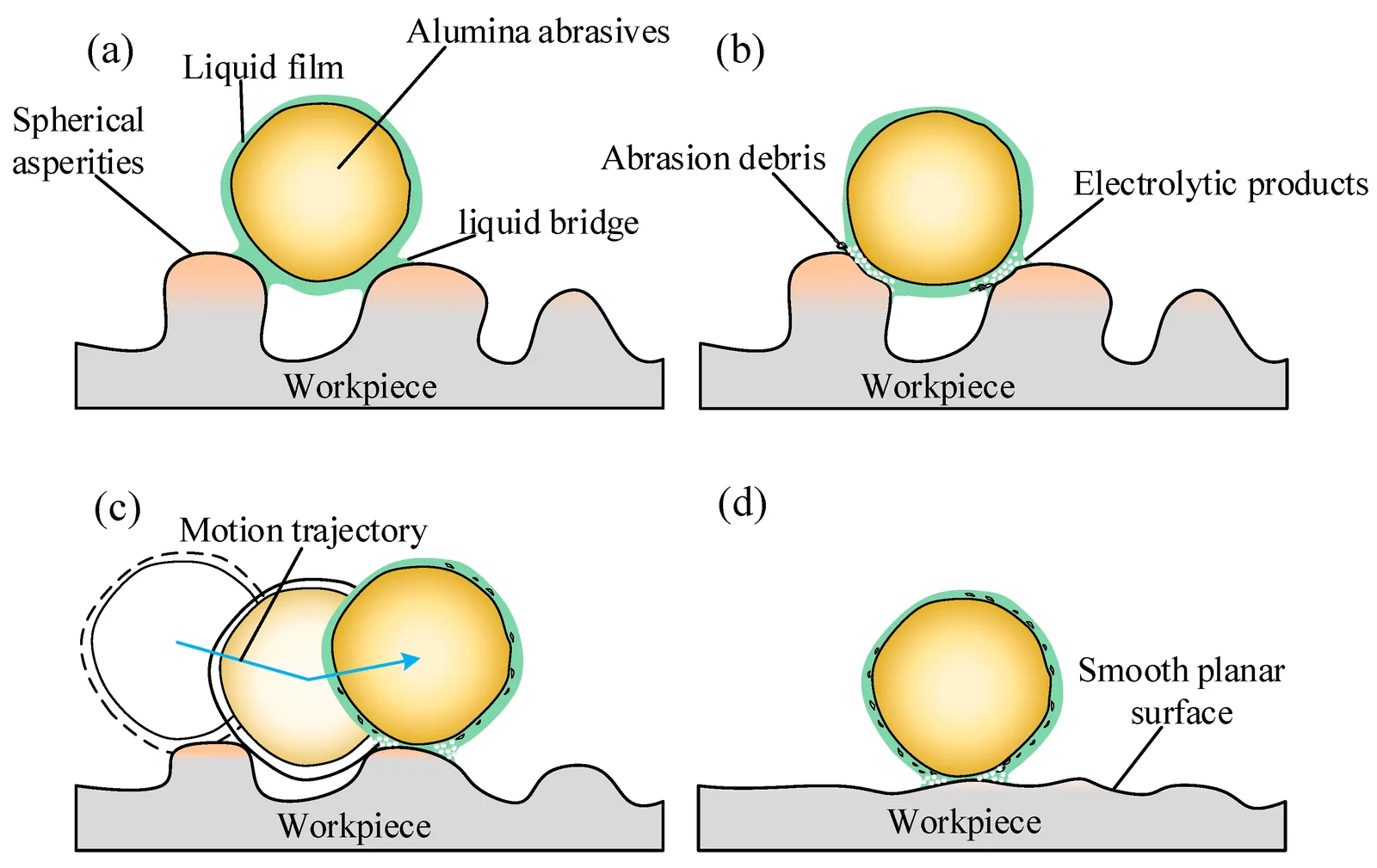
Schematic diagram of Q-DECMP and leveling mechanism (**a**) Initial contact of liquid film and liquid bridge; (**b**) electrochemical-mechanical dissolution of convex peaks; (**c**) abrasive scraping to renew the surface; (**d**) dynamic equilibrium of electrochemical-mechanical leveling.

The primary prerequisite for achieving planarization in the Q-DECMP process is selective contact. As shown in Figure 17a, alumina abrasive grains with a diameter of approximately 2 mm serve as the electrolyte carrier. Because the millimeter-scale abrasive size is much larger than the typical peak-to-valley roughness height of the SLM-TC4 titanium alloy initial surface (initial Rz = 47.3 ± 6.9 μm), the abrasive grains cannot fully conform to the concave areas of the workpiece surface. Simultaneously, due to the use of a high-viscosity, low-volatility NaCl-EG electrolyte, when a small amount of electrolyte is mixed with the abrasive grains, and relying on capillary force and surface tension between the grains, the electrolyte can form a uniform thin conductive liquid film on the surface of the abrasive grains, and a stable liquid bridge is established at the local contact area between the grains and the workpiece. During the machining process, the abrasive grains carrying the conductive liquid film effectively become “moving micro-electrodes.” Due to the uneven surface features, the liquid film and liquid bridge can only make physical contact with the peaks of the SLM surface (including spheroidized particles, banded protrusions, etc.). In contrast, in the concave and valley regions, the size effect of the abrasive grains prevents the establishment of a reliable conductive liquid bridge, resulting in a near-zero current density in those areas and effectively inhibiting electrochemical reactions. This special physical contact state, characterized by “peak contact and valley insulation,” forms the basis for the selective and localized material removal in the Q-DECMP process.

The second critical phase of the planarization process is the material removal from the peak regions, as illustrated in Figure 17b,c. Once a local current path is established between the abrasive grain liquid film and the peak regions, a significant anodic dissolution reaction occurs in the peak areas under the applied electric field (e.g., optimized 200 V voltage). Metal atoms on the surface of the TC4 titanium alloy are oxidized to form metal cations. However, unlike traditional immersion-type electrochemical polishing, the mechanical friction effect is indispensable in the Q-DECMP system. Under the relative motion between the electrodes (counter-rotation of the workpiece and polishing tank at a linear velocity of 1.0 m/s), the 2 mm alumina abrasive grains roll, press, and rub against the workpiece surface (Figure 17c). This mechanical action can scrape off, in real time and in situ, the loose product layer generated on the peak surfaces by the anodic reaction, thereby exposing fresh TC4 substrate to the electrolyte again. This exposure of fresh substrate further intensifies the local current density, promoting more efficient anodic dissolution. This process forms a dynamic cycle of “dissolution–friction removal–re-dissolution.” This synergistic mechanism prevents the severe accumulation of electrolysis products or non-selective over-corrosion caused by electrochemical action, and also compensates for the deficiency of pure mechanical polishing (as shown in Figure 8), which struggles to effectively remove peaks formed by plastic deformation. Ultimately, efficient layer-by-layer reduction is achieved, and the surface gradually becomes planar (as shown in Figure 17d).

In summary, a proposed planarization mechanism of Q-DECMP is initiated by the “localized contact of millimeter-scale abrasive grain liquid films/liquid bridges.” Through the high-frequency dynamic synergy of “electrochemical anodic dissolution and mechanical friction scraping,” it achieves the layer-by-layer reduction of spheroidized particles and a step effect on the SLM-formed surface. During this process, the electrochemical action ensures removal efficiency and valley protection, while mechanical friction maintains interfacial activity and removes reaction products [39]. This localized electrochemical–mechanical coupling behavior is consistent with previous studies on localized electrochemical removal of titanium alloys [40]. The two actions mutually promote each other, ultimately achieving efficient, low-damage, and green planarization of the titanium alloy surface with minimal electrolyte consumption.

## 5. Conclusions

This paper addresses typical additive manufacturing defects on selective laser melting (SLM)-formed TC4 titanium alloy surfaces, such as spheroidized particles, step effect, and high roughness, and proposes a quasi-dry electrochemical mechanical polishing (Q-DECMP) method based on a NaCl-EG electrolyte. Through a systematic study of the effects of key parameters including electrolyte composition, machining voltage, electrolyte-to-abrasive mass ratio, and linear velocity on surface quality and material removal efficiency, the following main conclusions are drawn:

(1) The electrolyte composition significantly influences the passivation and dissolution behavior of TC4 titanium alloy. The 0.5 mol/L NaCl-EG system forms a relatively dense passivation film, but its electrolytic dissolution activity is insufficient under high-voltage conditions; the 1 mol/L NaCl-H_2_O system exhibits a severe pitting tendency, resulting in poor surface integrity, whereas the 1 mol/L NaCl-EG system possesses both moderate electrical conductivity and quasi-passivation characteristics. XPS results indicate that the surface chemical state changes after Q-DECMP, suggesting that the surface reaction layer is modified during processing. Meanwhile, the electrochemical test results indicate that the 1 mol/L NaCl-EG system achieves a better overall balance between conductivity and surface quality, making it more suitable as the electrolyte for the Q-DECMP process.
(2) Under the conditions of a machining voltage of 200 V, an electrolyte-to-abrasive mass ratio of 1:20, a linear velocity of 1 m/s, and an inter-electrode gap of 6.5 mm, the overall polishing effect is near-optimal. After 40 min of processing, the surface roughness Ra of the sample is reduced from 6.89 μm to 0.77 μm, a reduction of approximately 88.8%, with an MRR of 612.5 μg/min. In comparison, purely mechanical machining (without applied voltage) can only reduce Ra to 4.98 μm, with an MRR of only 220 μg/min. The material removal efficiency of Q-DECMP is approximately 2.78 times that of pure mechanical machining, with apparent contributions of about 36% (mechanical) and 64% (electrochemical). This indicates that electrochemical dissolution is the dominant mechanism for leveling, while mechanical action mainly assists leveling and surface renewal; their synergy enables efficient leveling. The optimization criterion is surface roughness, and a multi-response optimization considering both MRR and Ra will be carried out in future work.
(3) Sequential surface morphology evolution analysis reveals a proposed selective planarization mechanism of Q-DECMP: alumina abrasive grains with a diameter of approximately 2 mm are much larger than the peak-to-valley undulation scale of the SLM surface (initial Rz = 47.3 ± 6.9 μm). The abrasive grains carrying the conductive liquid film preferentially contact the surface peak regions to form localized liquid bridges, while the electrochemical reactions in the concave pit regions are inhibited due to the lack of conductive pathways. Under the action of the electric field, anodic dissolution occurs in the peak regions, and mechanical friction simultaneously removes the loose reaction product layer, continuously exposing the fresh substrate, thereby forming a dynamic cycle of “dissolution-scraping-redissolution.” This achieves localized polishing with “preferential removal of peaks and effective protection of valleys.”
(4) The Q-DECMP process utilizes a thin-layer conductive liquid film on the surface of millimeter-scale alumina abrasive grains as the ion transport medium. In this work, only 40 g of NaCl-EG electrolyte was used for 800 g of alumina abrasives (electrolyte-to-abrasive mass ratio of 1:20), which is far less than the electrolyte volume required in conventional immersion polishing. This extremely low electrolyte consumption provides a low-cost route for the post-processing of additively manufactured metal parts. While maintaining high material removal efficiency, this method offers advantages in selective planarization and low electrolyte consumption. Subsequent research will focus on the applicability to complex curved surface components, long-term process stability, and feasibility for large-scale application.
(5) Limitations: Several limitations of this work should be noted. The process parameters were mainly investigated by a single-factor approach, and the interactions among voltage, electrolyte-to-abrasive mass ratio, and linear velocity were not systematically analyzed; only the voltage-linear velocity interaction was partially evaluated in Section 4.6. In addition, the current evolution with time was not systematically recorded, the build orientation and platform location were not varied, and only one batch of specimens was used. Furthermore, areal surface parameters such as Sa and Sz were not extracted because of the curved sidewall, and the detailed mechanism still requires further verification by in situ electrochemical measurements and interfacial adsorption characterization. Future work will focus on the multivariable optimization of the process parameters, evaluation of multiple independently fabricated specimens with different build orientations and platform locations, application to complex curved surfaces, and direct verification of the proposed mechanism.

## Figures and Tables

**Table 1:** TC4 powder characteristics and SLM process parameters.

Item	Value
Particle size distribution	15–53 μm (D10 = 19.4 μm, D50 = 36.9 μm, D90 = 59.7 μm)
Chemical composition (wt%)	Al 6.33, V 4.14, O 0.095, N 0.0071, H 0.0006
Layer thickness	0.04 mm
Fill laser power	240 W
Fill scanning speed	1300 mm/s
Contour laser power	180 W
Contour scanning speed	500 mm/s

**Table 2:** Electrolyte Composition.

Reagent Name	Density/(gcm^−3^)	Purity/%	Quality Score Percentage/%
NaCl	2.17	≥99.5%	5%
(CH_2_OH)_2_	1.1135	≥99.5%	95%

**Table 3:** Experimental conditions (Single-factor experimental design: when one parameter is varied, the other two parameters are kept constant.).

Items	Parameters	Notes
Workpiece size (mm)	Φ15 × 7	Fixed
Inter-electrode gap (mm)	6.5	Fixed
Al_2_O_3_ abrasive particle size (mm)	2.3	Fixed
NaCl mass fraction (Wt%)	5	Fixed
Weight ratio (electrolyte:abrasive)	1:10, 1:20, 3:40	Single-factor
linear velocity (m/s)	0.5, 1, 1.5	Single-factor
Voltage (V)	150, 175, 200, 225, 250	Single-factor

**Table 4:** Electrochemical parameters obtained from potentiodynamic polarization tests.

Electrolyte	*E_corr_* (V vs. SCE)	*I_corr_* (A)
1 mol/L NaCl-H_2_O	−0.691	1.8 × 10^−5^
1 mol/L NaCl-EG	−0.707	2.9 × 10^−6^
0.5 mol/L NaCl-EG	−0.658	2.6 × 10^−9^

**Table 5:** Changes in elemental atomic percentages before and after polishing as detected by EDS. Each value is the average of three measurements at different points on each surface (*n* = 3).

Element	Unpolished	Polished	Mechanical Polishing Alone
C (at%)	8.37	9.06	16.58
O (at%)	30.13	15.73	10.21
Al (at%)	6.16	6.76	6.51
Ti (at%)	53.10	65.71	63.93
V (at%)	2.24	2.74	2.77
Total	100	100	100

## Data Availability

The raw experimental data supporting the findings of this study are available on request from the corresponding author.
